# A rationally designed nanoparticle for RNA interference therapy in B-lineage lymphoid malignancies

**DOI:** 10.1016/j.ebiom.2014.10.013

**Published:** 2014-10-28

**Authors:** Fatih M. Uckun, Sanjive Qazi, Hong Ma, Lichen Yin, Jianjun Cheng

**Affiliations:** a Children's Center for Cancer and Blood Diseases, Children's Hospital Los Angeles (CHLA), Los Angeles, CA 90027, United States; b Division of Hematology-Oncology, Department of Pediatrics, University of Southern California Keck School of Medicine (USC KSOM), Los Angeles, CA 90027, United States; c Translational Oncology Program, Norris Comprehensive Cancer Center, University of Southern California Keck School of Medicine (USC KSOM), Los Angeles, CA 90027, United States; d Bioinformatics Program, Gustavus Adolphus College, 800 W College Avenue, St. Peter, MN 56082, United States; e Department of Materials Science and Engineering, University of Illinois at Urbana-Champaign (UIUC) Bioengineering Department, Urbana, IL 61801, United States

**Keywords:** Cationic polypeptides, Nanoparticles, Leukemia, Nanotechnology, Nanomedicine

## Abstract

The purposes of the present study were to further evaluate the biologic significance of the CD22ΔE12 molecular lesion and determine if it could serve as a molecular target for RNA interference (RNAi) therapy. We show that both pediatric and adult B-lineage lymphoid malignancies are characterized by a very high incidence of the CD22ΔE12 genetic defect. We provide unprecedented experimental evidence for a previously unrecognized causal link between CD22ΔE12 and aggressive biology of BPL cells by demonstrating that siRNA-mediated knockdown of CD22ΔE12 in primary BPL cells is associated with a marked inhibition of their clonogenicity. These findings provide the preclinical proof-of-concept that siRNA-mediated depletion of CD22ΔE12 may help develop effective treatments for high-risk and relapsed BPL patients who are in urgent need for therapeutic innovations. We also describe a unique polypeptide-based nanoparticle formulation of CD22ΔE12-siRNA as an RNAi therapeutic candidate targeting CD22ΔE12 that is capable of delivering its siRNA cargo into the cytoplasm of leukemia cells causing effective CD22ΔE12 depletion and marked inhibition of leukemic cell growth. Further development and optimization of this nanoparticle or other nanoformulation platforms for CD22ΔE12-siRNA may facilitate the development of an effective therapeutic RNAi strategy against a paradigm shift in therapy of aggressive or chemotherapy-resistant B-lineage lymphoid malignancies.

## Highlights

• CD22ΔE12 is a molecular target for RNA interference (RNAi) therapy in B-lineage lymphoid malignancies.
• A polypeptide-based hybrid nanoparticle formulation of CD22ΔE12-siRNA exhibits anti-leukemic activity.

## 1 Introduction

CD22, a member of the Siglec (sialic acid-binding Ig-like lectins) family of regulators of the immune system, is a negative regulator of multiple signal transduction pathways critical for proliferation and survival of B-lineage lymphoid cells. B-precursor acute lymphoblastic leukemia (BPL) is the most common form of cancer in children and adolescents. BPL cells express a dysfunctional CD22 due to deletion of Exon 12 (CD22ΔE12) arising from a splicing defect associated with homozygous intronic mutations (Uckun et al., 2010). CD22ΔE12 results in a truncating frame shift mutation yielding a mutant CD22ΔE12 protein that lacks most of the intracellular domain including the key regulatory signal transduction elements, such as the ITIMs that provide docking sites for the SH2 domains of SHP1, and all of the cytoplasmic tyrosine residues (Uckun et al., 2010). Our recent studies have demonstrated that CD22ΔE12 is a characteristic genetic defect of therapy-refractory clones in pediatric BPL and implicated the CD22ΔE12 genetic defect in the aggressive biology of relapsed or therapy-refractory pediatric BPL (Ma et al., 2012). Furthermore, forced expression of CD22ΔE12 in transgenic mice causes fatal BPL, demonstrating that CD22ΔE12 alone is sufficient as an oncogenic driver lesion for malignant transformation and clonal expansion of B-cell precursors (Uckun et al., unpublished data, 2014).

The purposes of the present study were to evaluate the biologic significance of the CD22ΔE12 molecular lesion in BPL and determine if it could serve as a molecular target for RNA interference (RNAi) therapy. Here we report that B-lineage lymphoid malignancies in children and adults are characterized by a high incidence of the CD22ΔE12 genetic defect. We further report a previously unrecognized causal link between CD22ΔE12 and aggressive biology of BPL cells by demonstrating that siRNA-mediated knockdown of CD22ΔE12 in primary BPL cells is associated with a marked inhibition of their clonogenicity. We also describe a unique polypeptide-based nanoparticle formulation of CD22ΔE12-siRNA as a first-in-class RNAi therapeutic candidate targeting CD22ΔE12 that is capable of delivering its siRNA cargo into the cytoplasm of leukemia cells causing effective CD22ΔE12 depletion and marked inhibition of leukemic cell growth. Further development and optimization of this nanoparticle may facilitate the development of an effective therapeutic RNAi strategy against a paradigm shift in therapy of aggressive or chemotherapy-resistant B-lineage lymphoid malignancies.

## 2 Methods

### 2.1 Gene Expression Profiling of Murine BPL Cells

Mouse leukemia cells were isolated from markedly enlarged spleens of CD22ΔE12-Tg, BCR-ABL Tg or Eμ-MYC Tg mice, splenocytes from wildtype healthy C57BL/6 mice served as controls. Total cellular RNA was extracted from homogenized lysate samples of leukemia cells using the Qiagen RNeasy Plus Mini Kit (catalog no. 74134) (Qiagen, Santa Clarita, CA). In order to minimize the genomic DNA contamination in the RNA samples, the homogenized lysate samples were first run through a DNA binding spin column prior to steps of RNA binding, washing and elution. Total RNA (45 μL/sample) was obtained with a concentration range of 60–200 ng/µL and an absorbance ratio (A260/A280) ranging from 1.9–2.1. RNA quality as judged by the integrity of the 28S and 18S ribosomal RNA was determined by an Agilent 2100 Bioanalyzer to ensure that it was acceptable for subsequent microarray analysis. Gene expression values for splenocytes from WT healthy C57/BL/6 mice (N = 4), leukemia cells from CD22ΔE12-Tg mice (N = 2), leukemia cells from BCR-ABL Tg mice (N = 2), and leukemia cells from Eμ-MYC Tg mice (N = 2) were estimated from RMA normalization of signal values following hybridization to the Affymetrix Mouse Gene 1.0 ST Array (1,102,500 probes, 35,512 genes) (accession #s: GSE58874 and GSE58872). PM signal values for probesets were extracted utilizing raw CEL files matched with probe identifiers obtained from a CDF file (MoGene-1_0-st-v1,r3.cdf obtained from http://www.aroma-project.org/vignettes/GeneSTArrayAnalysis) implemented by Aroma Affymetrix statistical packages run in the R-studio environment (version 0.97.551, R-studio Inc., running with R 3.01). The PM signals were quantified using Robust Multiarray Analysis (RMA) in a 3-step process including RMA background correction, quantile normalization, and summarization by a log additive model of probes in a probeset across these samples (RmaPlm method adapted in Aroma Affymetrix). All expression values were log_2_ scaled. The RLE (Relative Log Expression) and NUSE (Normalized Unscaled Standard Error) plots were utilized to assess array quality. For each gene and each array, ratios were calculated between the expression of a gene and the median expression of this gene across all arrays of the experiment. Box plots of the RLE and NUSE were within the quality bounds for all the arrays. Transcript cluster annotations for the mouse array were obtained from NetAffx website (http://www.affymetrix.com/Auth/analysis/downloads/na33/wtgene-32_2/MoGene-1_0-st-v1.na33.2.mm9.transcript.csv.zip).

### 2.2 Phosphoproteome Analysis Using Antibody Microarrays

The Phospho Explorer Antibody Array (Full Moon BioSystems, Inc., Sunnyvale, CA) consists of 1330 duplicate spots interrogating 1318 proteins (phosphorylated and unphosphorylated), housekeeping proteins (beta actin, GAPDH), negative controls (N = 4), empty spots (N = 4) and positive markers (N = 2). The antibodies are printed on standard-size coated glass slides and can be scanned on all microarray scanners that are compatible with 76 × 25 × 1 mm (3 in. × 1 in. × 1 mm) slides. The antibody array analyses were performed on cell lysate protein samples according to standard protocols. In brief, the cell lysates were biotinylated for 2 h at room temperature using the Biotin reagent in the antibody array assay kit (Full Moon BioSystems, Inc.). The array slide was blocked with blocking solution for 30 min at room temperature on an orbital shaker and then rinsed ten times with Milli-Q grade water. The biotinylated proteins (~ 80 μg) were mixed with 6 mL of coupling solution (Full Moon Biosystems, Inc.) and then added over the array slide for a 2-hour incubation at room temperature while shaking. The array was then washed with 30 mL of 1× Wash Solution (Full Moon Biosystems, Inc.) for 10 min for a total of three times and then rinsed ten times with Milli-Q water before detection of bound biotinylated proteins using Cy3-conjugated streptavidin. Subsequently, the array slide was incubated in Cy3-Streptavidin solution for 30 min in the dark while shaking. Next, the slide was washed with 1× Wash buffer and rinsed with Milli-Q water as before. The slide was then dried with compressed nitrogen and scanned using Axon GenePix 4000 scanner and the images were analyzed with GenePix Pro 6.0 (Molecular Devices, Sunnyvale, CA). The fluorescence signal of each antibody was obtained from the fluorescence intensity of this antibody spot after subtraction of the blank signal (spot in the absence of antibody). Two technical replicates were performed for each sample. Median signal intensity for each spot was extracted from array images and the average intensity for each antibody reaction with the protein was determined for replicate spots. Data were normalized utilizing the median intensity values for the 1318 antibodies on each array (normalized data = average signal intensity of replicate spots / median signal). The normalized data were log_10_ transformed and mean centered to the WT samples (N = 4, 2 technical replicates for each of 2 samples) (Accession #s: GSE58873 and GSE58874). Student 's T-tests (unequal variance correction, Microsoft Excel) compared mean-centered, log_10_ transformed protein expression levels in BPL cells from CD22ΔE12-Tg mice vs. splenocytes from WT mice and BPL cells from BCR-ABL Tg or MYC Tg mice. 142 phosphoproteins were differentially expressed with P-values < 0.02 (false discovery rate = 18%; 99 of these proteins were downregulated and 43 were upregulated). Most significant effect sizes were compiled by filtering according to fold changes greater than 1.1 and P < 0.02.

### 2.3 CD22ΔE12-driven Gene Expression Cassette

32 genes (represented by 43 probesets) encoding the 34 most significantly overexpressed proteins (including 2 phosphorylated forms of JunB (JunB (Phospho-Ser259) and JunB (Phospho-Ser79))); and NFκB (NFκB-p105/p50 (Ab-932), NFκB-p65 (Ab-435))), in CD22ΔE12-Tg mice were identified by cross referencing the Swiss Prot ID provided by the manufacturers of the antibody array with the Affymetrix mogene10 annotation data provided by Bioconductor for R (mogene10sttranscriptcluster.db file) as well as the conversion tool provided by MGI, Jackson Laboratory (http://www.informatics.jax.org/homology.shtml). These mouse probesets were compared for differential expression in 2 CD22ΔE12 mice versus 4 Wildtype mice utilizing a Mixed Model ANOVA (lme4 package from R; 2 fixed factors for “probeset” and “genotype” (CD22ΔE12 and WT), 1 interaction term for “probeset × genotype”, and 1 random factor for mouse ID) to identify significantly affected mRNA levels (least square means and standard error estimates calculated from the interaction term parameters) for the significantly affected proteins identified by the antibody array. The mouse gene signature differentially expressed in CD22ΔE12-Tg mouse BPL cells comprised of 11 significantly affected probesets representing 10 upregulated genes and one downregulated gene. In order to examine the representation of this CD22ΔE12-dictated gene expression signature in human BPL cells, we first identified the human orthologs on the Affymetrix Human Genome U133 Plus 2.0 Array (hgu133plus2.db file) downloaded from the Bioconductor repository Affymetrix by cross-referencing the hugene10 annotation data (hugene10sttranscriptcluster.db file) provided by the Bioconductor repository for R software (http://www.bioconductor.org/) as well as the conversion tool provided by MGI, Jackson Laboratory (http://www.informatics.jax.org/homology.shtml). The CD22ΔE12-driven gene expression cassette comprised of the 10 upregulated genes in mouse BPL cells was represented in the transcriptome of human BPL cells by 25 probesets for the human orthologs of the up regulated 10 signature genes. We compared the expression of these genes in normal hematopoietic cells (N = 74, GSE13159) versus primary leukemia cells BPL patients (N = 76, GSE28460, GSE18497), normal lymphoid cells (N = 130; GSE10846, GSE12195, GSE12453, GSE13159) versus primary leukemia cells from CLL patients (N = 578; GSE13159, GSE39671) and normal lymphoid cells (N = 130) versus primary neoplastic cells from mantle cell leukemia/lymphoma patients (MCL; N = 38, GSE36000) utilizing the RMA normalized database containing these subsets of adult and pediatric leukemias. Mixed Model ANOVAs (2 fixed factors for “probeset” and “diagnostic group”, 1 interaction term for “probeset × diagnostic group” and 1 random factor for sample ID) were constructed for each comparison to assess probeset level significance (least square means and standard error estimates obtained from the interaction term parameters) and significance of the mouse gene signature as a whole for each of the normal versus human leukemic group comparisons (least mean squares and standard error estimates obtained from the “diagnostic group” factor).

### 2.4 Determination of CD22ΔE12-index Values by Multiprobe Gene Expression Profiling

We compiled archived transcriptome profiling datasets in the Human Genome U133 Plus 2.0 Array platform regarding gene expression levels in primary leukemia cells from pediatric high-risk BPL patients and adult patients with chronic lymphocytic leukemia [CLL], mantle cell leukemia/lymphoma [MCL], or Burkitt's lymphoma (BL). To enable comparison of samples across studies, 2 sets of normalization procedures were performed that merged the raw data. Perfect Match (PM) signal values for probesets were extracted utilizing raw CEL files matched with probe identifiers obtained from the Affymetrix provided CDF file (HG-U133_Plus_2.cdf) implemented by Aroma Affymetrix statistical packages ran in R-studio environment (version 0.97.551, R-studio Inc., running with R 3.01). The PM signals were quantified using Robust Multiarray Analysis (RMA) in a 3-step process including RMA background correction, quantile normalization, and summarization by Median Polish of probes (RMA method adapted in Aroma Affymetrix). RMA background correction estimates the background by a mixture model whereby the background signals were assumed to be normally distributed and the true signals are exponentially distributed. Normalization was achieved using a two-pass procedure. First the empirical target distribution was estimated by averaging the (ordered) signals over all arrays, followed by normalization of each array toward this target distribution.

BLAT analysis on CD22 probe sequences for probeset 217422_s_at (covering exons 10–14) deposited in the Affymetrix NetAffx™ Analysis Center (http://www.affymetrix.com/analysis/index.affx) was mapped onto specific CD22 exons and visualized using the UCSC genome browser (http://genome.ucsc.edu/cgi-bin/hgBlat?command=start). This analysis was designed to locate ≥ 25-bp long sequences with ≥ 95% similarity in the entire genome. The BLAT-based exon designations according to 3 reference sequences (viz.: UCSC genes, Ensembl gene predictions, Human mRNA Genbank) were as follows: HG-U133_PLUS_2:217422_S_AT_11 aligned to chr19: 35837525–35837549 (Exon 14); HG-U133_PLUS_2:217422_S_AT_10 aligned to chr19: 35837478–35837502 (Exon 14); HG-U133_PLUS_2:217422_S_AT_8 aligned to chr19: 35837100–35837124 (Exon 13); HG-U133_PLUS_2:217422_S_AT_9 aligned to chr19: 35837117–35837139 (Exon 13); HG-U133_PLUS_2:217422_S_AT_7 aligned to chr19:35836590–35836614 (Exon 12); HG-U133_PLUS_2:217422_S_AT_6 aligned to chr19:35836566–35836590 (Exon 12); HG-U133_PLUS_2:217422_S_AT_5 aligned to chr19:35836535–35836559 (Exon 12); HG-U133_PLUS_2:217422_S_AT_4 aligned to chr19:35835979–35836003 (Exon 11); HG-U133_PLUS_2:217422_S_AT_3 aligned to chr19:35835811–35835960 (Exon 11); HG-U133_PLUS_2:217422_S_AT_2 aligned to chr19:35835741–35835765 (Exon 10). Background corrected and RMA-normalized signal values for each probe in each sample were log_2_ transformed and median centered across 11 probes per sample. An Exon 12 Index was calculated by subtracting the median centered expression values of the Exons 10–11 plus Exons 13–14 probes from the median centered expression values of the Exon 12 probes. The CD22ΔE12 index values were calculated and compared for, (i) normal bone marrow hematopoietic cells vs. primary leukemia cells from high-risk BPL patients, including multi-lineage leukemia gene rearranged (MLL-R^+^) (N = 95; GSE11877, GSE13159, GSE13351), BCR-ABL^+^ (N = 123; GSE13159, GSE13351) and E2A-PBX1^+^ patients (N = 61; GSE11877, GSE13159, GSE13351), (ii) and adult leukemia/lymphoma cells from patients with chronic lymphocytic leukemia (CLL) (N = 578; GSE13159, GSE39671), mantle cell lymphoma/leukemia (MCL) (N = 38; GSE36000) and Burkitt's lymphoma/leukemia (BL) (N = 5; GSE12453) vs. normal B-cells (N = 130; GSE10846, GSE12195, GSE12453, GSE13159). To compare the incidence of CD22ΔE12 in subsets of patients the proportion of leukemic samples below the lower 95% Confidence interval for normal non-leukemic cells vs. leukemia/lymphoma cells for each patient sub-population was determined. One-way ANOVA followed by Dunnett's post hoc method was utilized to calculate P-values of the comparisons. Dot plots of the sample level CD22ΔE12-index values depicted the proportion of expression values below the lower 95% confidence intervals for the normal samples.

### 2.5 RT-PCR Analysis of Human Leukemia Cells for CD22ΔE12 mRNA Expression

Reverse transcription (RT) and polymerase chain reaction (PCR) were used to examine expression levels of wildtype CD22 and CD22ΔE12 transcripts in human leukemia cells, as previously described (Uckun et al., 2010, Ma et al., 2012). Total cellular RNA was extracted from ALL cells using the QIAamp RNA Blood Mini Kit (catalog no. 52304) (Qiagen, Santa Clarita, CA, USA) following the manufacturer's instructions. Oligonucleotide primers were obtained from Integrated DNA Technologies (IDT, San Diego, CA, USA). The P7 primer set (Fwrd: 5′-GCCAGAGCTTCTTTGTGAGG-3′ and Rev: 5′-GGGAGGTCTCTGCATCTCTG-3′) amplifies a 182-bp region of the CD22 cDNA extending from Exon 11 to Exon 13 and deletion of Exon 12 results in a smaller CD22ΔE12-specific PCR product of 63-bp size using this primer set. The P10 primer set was used as a positive control to amplify a 213-bp region of the CD22 cDNA in both wildtype CD22 and CD22 with ΔE12 (Fwrd: 5′-ATCCAGCTCCCTCCAGAAAT-3′ and Rev: 5′-CTTCCCATGGTGACTCCACT-3′). Qiagen One-Step RT-PCR Kit (catalog no. 210212) (Qiagen, Santa Clarita, CA, USA) was used following manufacturer's instructions to amplify the target PCR products. The conditions were 1 cycle (30 min 50 °C, 15 min 95 °C) and 35 cycles (45 s 94 °C, 1 min 60 °C, 1 min 72 °C). PCR products were separated on a 2% agarose gel containing ethidium bromide. Gel images were taken with an UVP digital camera and a UV light in an Epi Chemi II Darkroom using the LabWorks Analysis software (UVP, Upland, CA).

### 2.6 Leukemia Cells

We used 3 ALL xenograft clones derived from spleen specimens of xenografted NOD/SCID mice inoculated with leukemia cells from pediatric BPL patients. The secondary use of leukemic cells for subsequent laboratory studies did not meet the definition of human subject research per 45 CFR 46.102 (d and f) since it did not include identifiable private information, and it was approved by the IRB (CCI) at the Children's Hospital Los Angeles (CHLA) (CCI-10-00141; CCI review date: 7-27-2010; IRB approval: 7-27-2010). Human Subject Assurance Number: FWA0001914. We also used the ALL-1 (Ph^+^ adult BPL), DAUDI (Burkitt's lymphoma/B-cell ALL) and RAJI (Burkitt's lymphoma/B-cell ALL) cell lines.

### 2.7 Colony Assays

The effects of the transfections with CD22ΔE12-siRNA vs. scrambled (scr)-siRNA, treatments with nanoformulations of CD22ΔE12-siRNA vs. scr-siRNA on BPL xenograft clones or the CD22ΔE12^+^ leukemia cell lines were examined using in vitro colony assays as described (Uckun et al., 1987). Cells were treated with the respective reagents for 24 h at 37 °C. Immediately after transfection or completion of the 24 h treatments, cells (0.5 × 10^6^ cells/mL) were suspended in RPMI supplemented with 0.9% methylcellulose, 30% fetal calf serum, and 2 mM l-glutamine. Controls included untreated cells. Duplicate–quadruplicate 1 mL samples containing 0.5 × 10^6^ cells/sample were cultured in 35 mm Petri dishes for 7 d at 37 °C in a humidified 5% CO_2_ atmosphere. On day 7, colonies containing ≥ 20 cells were counted using an inverted Nikon Eclipse TS100 microscope. For colony assays of primary leukemia cells, we substituted RPMI with alpha-MEM and FBS with calf bovine serum. Colony images were taken using a Digital Sight DS-2MBW Nikon camera.

### 2.8 Preparation of a Human Full-length CD22 and CD22ΔE12–14 Plasmids

The cDNA fragments encoding either full-length CD22 (CD22FL) or truncated CD22 lacking exons 12–14 (CD22ΔE12–14) were generated by PCR amplification using the Phusion High Fidelity PCR Kit (New England Biolabs, catalog no. E0553L) with the following primer sets: full-length Fwd/Rev 5′-CTTGGTGCTAGCATGCATCTCCTCGGC-3′/5′CCGGTCTCGAGGATGTTTGAGGATCACATAGTC-3′, and truncation ΔE12–14 Fwd/Rev 5′-CTTGGTGCTAGCATGCATCTCCTCGGC-3′/5′-CCGGTCTCGAGCCTTTTTATTCCTCAC-3′. The correct PCR products (FL: 2541-bp and ΔE12–14: 2208-bp) were ligated into the 8497-bp lentiviral vector pCL6-2AEGwo through the NheI and XhoI restriction sites (underlined) using the Quick Ligase kit (New England Biolabs catalog no. M2200L) following the manufacturer's instructions. The pCL6-2AEGwo lentiviral backbone vector, a kind gift from Dr. Zanxian Xia, School of Biological Science and Technology, Central South University, Changsha, Hunan 410078, China, contains both a “ribosome-skip” fragment encoding the 2A-like peptide APVKQTLNFDLLKLAGDVESNPGP and an in-frame *eGFP* fluorescent coding sequence downstream of a multiple cloning site. The expression of GFP allows transduced cells to be identified using fluorescent microscope. The correct clones were first confirmed through restriction enzyme digestion and then characterized by sequencing the backbone-insert junctions on both 5′ (83-bp upstream) and 3′ (74-bp downstream) ends with the forward primer 5-CAGCCTGCTTCTCGCTTCTGTT-3′ and the reverse primer 5′-CTCCTGCCAACTTGAGAAGGTC-3′ performed by GENEWIZ, Inc. (South Plainfield, NJ) using Applied Biosystems BigDye version 3.1. In this procedure, 3′-fluorescent-labeled dideoxynucleotides (dye terminators) were incorporated into DNA extension products (cycle sequencing). The resulted DNA sequences were analyzed by BioEdit v7.2.0 (http://www.mbio.ncsu.edu/bioedit). Plasmids with confirmed inserts were transformed to and propagated in chemical competent DH5α strain of *Escherichia coli* cells. Milligrams of plasmid DNA was extracted and purified from an approximately 100 mL of the bacterial culture using Invitrogen PureLink® HiPure Plasmid Filter Maxiprep Kit (catalog no. K2100-17) following manufacture's instruction. Plasmid DNA quality and quantity was determined using Thermo Scientific NanoDrop 2000 spectrophotometer. For the generation of infectious lentiviral particles, the pCL6-2AEGwo lentiviral vectors containing either the CD22 FL or CD22ΔE12–14 expression cassettes were co-transfected with the ViraPower™ packaging plasmid mixture (K4975-00, Invitrogen) into 293T cells using the BioT™ transfection reagent (BioLand Scientific, CA). Supernatants of 293T cells were harvested 72 h later, filtered (pore size, 0.22 μm), and viral particles were concentrated by ultracentrifugation (120,000 ×*g* for 3 h at 4 °C). The supernatants were discarded; the remaining viral pellets were resuspended in 100 μL OptiMEM by gentle pipetting, aliquoted, and stored at − 80 °C until use. Infectious titers of the *Lentivirus* stocks were determined by biological titration, in which 293T cells in a 24-wells plate were transduced with serial dilutions (1:10, 1:100, 1:1000) of the *Lentivirus* stock. Titers of all viral stocks were equalized by adjusting the concentration of viral particles to 5 × 10^6^ transduction units (t.u.)/mL. For transduction of leukemia cells, 1 × 10^5^ cells at log growth phase were incubated with lentiviral particles (20 μL) and 2 μL polybrene (0.1 mg/mL) (Sigma-Aldrich) in a final volume of 100 μL for 3 h. Transduced cells were examined for colony formation in a semi-solid methylcellulose culture system to determine the effects of transduction on their in vitro clonogenicity (number of colonies/105 cells plated) and proliferative activity (number of cells/colony), as described (Uckun et al., 1987).

### 2.9 Animal Research Approval

The animal research in mice was conducted according to Institutional Animal Care and Use Committee (IACUC) Protocols 280–12 and 293–10 that were approved by the IACUC of CHLA. All animal care procedures conformed to the Guide for the Care and Use of Laboratory Animals (National Research Council, National Academy Press, Washington DC 1996, USA).

### 2.10 Construction of pEμ-SR-R3Y Yeast Cloning Vector and pEμ-SR-CD22ΔE12 Transgenic Expression Vector

We established a new CD22ΔE12 transgenic mouse model using a novel transgenic expression vector designated ‘pEμ-SR-CD22ΔE12’ (accession # LM652705). The pEμ-SR backbone plasmid (Gabrielson et al., 2012, Lu et al., 2011) containing the mouse IgH enhancer (Eμ) and the potent SRα mammalian promoter (Yin et al., 2013a) along with SV40 polyA termination sequences was kindly provided by Dr. Jerry Adams (WEHI, Melbourne, Australia). Pronuclear microinjection of the human CD22ΔE12 transgene, founder generation, and genotyping analysis of tail DNA were performed under a service contract at the UC Davis Mouse Biology Program using standard methods. The 5.3-kb [promoter — CD22ΔE12 cDNA-poly A] fragment of the pEμ-SR-CD22ΔE12 transgene construct was microinjected into the pronuclei of freshly fertilized oocytes from C57BL/6J mice. Injected oocytes were transferred to day 0.5 postcoitus (dpc) pseudopregnant CD-1/Crl females to generate CD22ΔE12-Tg mice. Founders were mated to C57BL/6J mice obtained from Jackson Laboratories; CD-1/Crl mice were obtained from Charles River laboratories. All mouse procedures were carried out in accordance with the Institutional Animal Care and Use Committee at the University of California, Davis (IACUC #15723). Tg founder mice were identified by PCR analysis of genomic tail DNA. Tg male mice were crossed to age-matched wildtype female C57BL/6 mice to produce transgenic lines and pups were screened for the presence of the transgene by PCR analysis of tail-extracted DNA. We also performed RT-PCR to confirm the expression of the CD22ΔE12 transgene transcript in splenocytes from pre-leukemic CD22ΔE12-Tg mice as well as BPL cells isolated from leukemic CD22ΔE12-Tg mice by using a 5′ CD22ΔE12 E10 primer (E10-F) and a 3′ vector backbone primer (CD22ΔE12Tg-R2) to amplify a segment of the transgene message spanning CD22ΔE12 exons 10, 11, 13, and 14. Total RNA was extracted from splenocytes of wild type and CD22ΔE12-Tg mice using the RNeasy Plus Mini Kit (catalog no. 74134, Qiagen, CA). A semi-quantitative RT-PCR reaction was carried out where the first-strand cDNA was synthesized using SuperScript III (catalog no. 18080-051, Invitrogen CA) and, the second PCR reaction was performed using the Phusion High-Fidelity PCR Master Mix Kit (New England Biolabs) and the following thermal cycling conditions: initial denaturation 98 °C × 30 s, 35 cycles of denaturation 98 °C × 10 s, annealing 60 °C × 10 s and extension 72 °C × 30 s. The primers that specifically detect the 3′ end of CD22ΔE12 transgene were: Fwd: E10-F: ATCCTCATCCTGGCAATCTG and Rev: CD22ΔE12Tg-R2: 5′-CACCACCTTCTGATAGGCAGCC-3′ (estimated amplicon size: 436-bp). To ensure the quality and quantity of the 1st strand cDNA, a fragment of the mouse β-Actin cDNA was also amplified yielding a 206-bp product with primers Fwd 5′-CCTCTATGCCAACACAGTGC-3′, Rev 5′-CCTGCTTGCTGATCCACATC-3′.

### 2.11 Control Eμ-Myc and BCR-ABL Tg mice

Eμ-Myc Tg male founders (B6.Cg-Tg [IgHMyc] 22Bri/J, hemizygous for Tg [IgHMyc] 22Br, JAXEAST: AX12) were obtained from the Jackson Laboratory (Bar Harbor, Maine). These mice were bred to wildtype female C57BL/6J mice to establish a colony of Eμ-Myc transgenic mice (Harris et al., 1988). For genotyping of Eμ-Myc Tg mice, DNA was extracted from approximately 3 mm tail snips using Qiagen DNEasy blood and tissue kit following the manufacturer's instructions. DNA was then screened for the Myc transgene via TaqMan® qPCR via the relative Ct method multiplexed with the following oligos: Transgenic target primers/probe include a forward primer ATGTGCGCGGAACCCCTATT, reverse primer GGGCGACACGGAAATGTTG, and probe 6Fam-ACATTCAAATATGTATCCGCTCATGAGACA-NFQ and were quantified next to endogenous reference primers/probe including a forward primer GTCATCAAGTGAGAAAGACATCCT, reverse primer CATCATGAATTTTGATAAGCCCATT, a TaqMan probe CTCCTGGCTGCCTGGCTGGC with a 5′ VIC label (4,7,2′-trichloro-7′-phenyl-6-carboxyfluorescein) as fluorescence reporter and a 3′ TAMRA label (6-carboxytetramethylrhodamine) as quencher. Briefly, 2 μL of each DNA sample was tested in a total reaction volume of 10 μL utilizing Qiagen QuantiTect PCR kit with ROX chemistry. 384 plates were processed on an AB7900HT with the following cycles: 95 °C for 15 min and then 40 cycles of 95 °C for 30 s, and 60 °C for 1 min. Each DNA sample ΔCt was compared against the ΔCt of a transgenic mutant animal as well as a transgenic negative (wildtype) DNA sample using AB RQ Manager 1.2.1 software. Likewise, commercial BCR-ABL Tg [B6.Cg-Tg(BCR/ABL)623Hkp/J] mice were also obtained from the Jackson Laboratory. The BCR-ABL Tg mice contain the truncated murine metallothionein-1 promoter driving the expression of the 190-kDa BCR-ABL fusion protein characteristic of the t(9;22) BPL. These mice were bred to wildtype female C57BL/6J mice to establish a colony of BCR-ABL transgenic mice. Pups were screened for the presence of the BCR-ABL transgene by PCR analysis of tail DNA with a BCR-ABL Tg forward primer AGAGATCAAACACCCTAACCT and a BCR-ABL Tg reverse primer CCAAAGCCATACTCCAAATGC for an expected BCR-ABL Tg amplicon of 417-bp. Both transgenic mouse lines develop fatal BPL. Leukemic splenocytes from these commercial transgenic mice that developed fatal leukemia with massive splenomegaly were isolated at the time of the necropsy and subjected to gene expression profiling and phosphoprotein profiling in side by side comparison with CD22ΔE12-Tg BPL cells.

### 2.12 RNAi knockdown of CD22ΔE12 expression

CD22ΔE12-siRNA has been designed to specifically target the Exon 11 and Exon 13 junction of the CD22ΔE12 mature mRNA produced by aberrant splicing of the CD22ΔE12 pre-mRNA with intronic mutations. The specific CD22ΔE12 S-oligos were synthesized and purified with a reverse phase cartridge at GeneLink (Hawthorne, NY). The CD22ΔE12-siRNA duplex (MW: 13,492) was custom synthesized by GeneLink as a single strand oligo and annealed after all deprotection and purification steps were completed. The segment of the mutant human CD22ΔE12 mRNA that stretches +/− 20-bp from the Exon 11–Exon 13 junction was used as a target sequence to generate potential siRNA candidates using an algorithm provided by siDESIGN Center (GE Dharmacon, Lafayette, CO) with default parameters settings. Three AA-motif (N_19_-mer) target (sense) sequences covering the breakpoint (underlined) were initially selected, these are: 5′-AAAGAGATGCAGAGTCCTC-3′, 5′-GAGATGCAGAGTCCTCAGA-3′, and 5′-AAGAGATGCAGAGTCCTCA-3′. Phosphorothioate modification (denoted as “*”) was implemented for the first and the last 3 nucleotides of the 5′ and 3′ ends, respectively, followed by addition of 3′ end TT overhang. The sequence of the CD22ΔE12 antisense S-oligo was 5′-G*A*G*GACUCUGCAUCUC*U*U*UTT-3′ and the sequence of the corresponding sense oligo in the siRNA duplex was 5′-A*A*A*GAGAUGCAGAGUC*C*U*CTT-3′. To document the specificity of the antisense S-oligos we used appropriate scrambled (scr) control S-oligos with the same modification custom-prepared by GeneLink (antisense 5′-A*C*G*UGACACGUUCGGA*G*A*ATT-3′; sense 5′-U*U*C*UCCGAACGUGUCA*C*G*UTT-3′). The transfection of ALL-1 cells and BPL xenograft cells with CD22ΔE12-siRNA and control/scrambled siRNA was accomplished using the Amaxa Cell Line Nucleofector Kit T (catalog no. VCA-1002, Lonza, Cologne, Germany) and a Nucleofector II device according to a protocol specifically designed for the transfection of human B-lineage lymphoid cells (VCA-1002/C005) following the manufacturer's recommendations. Approximately 2–5 × 10^6^ cells were transfected with 50 nM (unless otherwise indicated) CD22ΔE12-siRNA. Controls included untransfected cells and cells transfected with scr-siRNA. An aliquot of the cells was transferred to a 6-well plate with 1.5 mL pre-warmed RPMI culture medium then kept overnight at 37 °C. The transfection efficiency was confirmed to be > 50% by transfecting cells with Green transfection indicator or siGlo™ (Thermo Scientific Dharmacon, D-001630-01-05) and by counting the green fluorescent cells. RNAi knockdown of CD22ΔE12 expression was documented by RT-PCR analysis of RNA samples using the P7 (WT: 182 bp, Mutant: 63 bp) and P10 (213 bp, control) primer pairs after 48 h and Western blot analysis of whole cell lysates after 72 h, as described (Ma et al., 2012).

### 2.13 CD22ΔE12-siRNA nanoparticles

By using a 200-mer of the cationic cell-penetrating α-helical peptide poly(γ-(4-vinylbenzyl)-l-glutamate/PVBLG)-8 (Gabrielson et al., 2012, Lu et al., 2011) and previously published procedures for preparing PVBLG-8 based nanoformulations of siRNA (Yin et al., 2013a), we developed a nanoformulation of CD22ΔE12-specific siRNA. The negatively charged siRNA was complexed with the positively charged PVBLG-8 at the optimal weight ratio of 20 via electrostatic interactions (Gabrielson et al., 2012, Lu et al., 2011, Yin et al., 2013a). PVBLG-8 was synthesized via hexamethyldisilazane (HMDS)-initiated controlled ring opening polymerization of γ-(4-vinylbenzyl)-l-glutamate NCA (VB-l-Glu-NCA) followed by side-chain vinyl group oxidation to aldehyde and amination (Gabrielson et al., 2012, Lu et al., 2011, Yin et al., 2013a). Another polyanion (PAOBLG) (Zhang et al., 2011) was also incorporated at the optimal PAOBLG/siRNA weight ratio of 2 to further stabilize the nanostructure and strengthen the electrostatic interactions of the cationic PVLG-8 with the CD22ΔE12-siRNA. Cyanine dye Cy3 has a maximum excitation at 550 nm and emits maximally in the red end of the spectrum at 570 nm. We used 5′ Cy3-labeled CD22ΔE12-siRNA to prepare a PVLG-8 based CD22ΔE12-siRNA formulation for cellular uptake and trafficking experiments using confocal imaging. The Cy3-labeled CD22ΔE12-siRNA duplex was custom-prepared by Integrated DNA Technologies, Inc. (Coralville, Iowa). This material was used to prepare a CD22ΔE12-siRNA nanoparticle formulation (NPF) for cellular uptake and trafficking experiments using confocal imaging. The ability of the nanoparticles to knock down CD22ΔE12 expression was examined using RT-PCR (Ma et al., 2012) and their anti-leukemic activity against CD22ΔE12-positive human leukemia cell line ALL-1 was evaluated using colony assays (Uckun et al., 1987).

### 2.14 Physicochemical characterization of siRNA containing nanoparticles

Size measurement by the dynamic light scattering (DLS) technique was performed on a DynaPro Titan Instrument (Wyatt Technology Corp., Santa Barbara, CA) at the USC Nano BioPhysics Laboratory (Uckun et al., 2013). We used the Quant-IT RiboGreen RNA assay (Invitrogen) and a Synergy HT Biotek fluorescence microplate reader to measure the siRNA content of the PVLG-8 based nanoparticles. The presence of siRNA in the formulations was also confirmed in 2% agarose gels prepared using 0.5 μg/mL of ethidium bromide and 3% SDS that allows staining of the siRNA content with ethidium bromide. A 2% agarose gel was prepared using UltraPure Agarose and 0.5 μg/mL of ethidium bromide (Invitrogen, Grand Island, NY). The 7 × 10 cm gel was immersed in 1× TBE buffer and run in a horizontal electrophoresis system (Bio-Rad, Mini-Sub Cell GT cell) for 1 h at 60 V. We used the 1 kb Plus DNA ladder (Invitrogen) as size markers. Samples were prepared in RNase-Free water (Qiagen, Valencia, CA) and 6× DNA loading buffer (Bioland Scientific, Paramount, CA). After sample migration down the gel, gel images were taken with an UVP digital camera and UV light in an Epi Chemi II Darkroom using the LabWorks Analysis software (UVP, Upland, CA).

### 2.15 Confocal laser scanning microscopy

Subcellular localization of Cy3-labeled CD22ΔE12-siRNA delivered in the PVLGB-8 based hybrid nanoparticles was examined by immunofluorescence and spinning disk confocal microscopy using previously described procedures for slide preparation and imaging (Uckun et al., 2012). Cyanine dye Cy3 has a maximum excitation at 550 nm and emits maximally in the red end of the spectrum at 570 nm. After incubation with the PVBLG-8 based formulation of Cy3-labeled siRNA for 6 h, the internalized Cy3-labeled CD22ΔE12-siRNA was detected and localized using the tetramethyl rhodamine (TRITC) filter sets since the excitation and emission spectra are very close to those of TRITC. Slides were imaged using the PerkinElmer Spinning Disc Confocal Microscope and the PerkinElmer UltraView ERS software (Shelton, CT) or the Volocity V5.4 imaging software (PerkinElmer, Shelton, CT). The coverslips were fixed with ice-cold MeOH at − 20 °C for 10 min. Cells were stained with a mouse monoclonal anti-tubulin antibody (Sigma catalog no. T6199; Sigma-Aldrich, St. Louis, MO) for 1 h at room temperature. Cells were washed with PBS and incubated with green-fluorescent Alexa Fluor 488 dye-labeled goat anti-mouse IgG (secondary Ab) (catalog no.: A11001, Invitrogen, Carlsbad, CA) for 1 h. Cells were then washed with PBS and counterstained with the blue fluorescent DNA-specific nuclear dye 4′,6-diamidino-2-phenylindole (DAPI). The coverslips were inverted, mounted onto slides in Vectashield (Vector Labs, Burlingame, CA) to prevent photobleaching, and sealed with nail varnish. UltraCruz Mounting Medium containing 1.5 μg/mL of DAPI was purchased from Santa Cruz Biotechnology, Inc. (Santa Cruz, CA).

### 2.16 Immunofluorescence staining and flow cytometric analysis of ALL xenograft cells

A panel of commercially available monoclonal antibodies was used for immunophenotyping of leukemic cells from spleen specimens of NOD/SCID mice xenografted with primary human ALL cells by standard immunofluorescent staining and multiparameter flow cytometry as previously reported (Uckun et al., 2013). The antibodies were obtained from BD Biosciences (San Jose, CA) and included: HLA/DR/DP/DQ FITC: catalog no.: 555558, HLA-A,B,C Phycoerythrin: catalog no.: 555553, CD10 (APC) BD catalog no.: 340923, CD19 (APC-H7) clone: SJ25C1 BD catalog no.: 560177, CD34 (Per CP-Cy5.5) BD catalog no.: 347203, and CD45 (V450) clone: H130 BD catalog no.: 560367. The labeled cells were analyzed on a LSR II flow cytometer (Becton Dickinson, Lakes, NJ). Controls included unstained cells as well as cells that were stained with a cocktail of control mouse IgG labeled with PE, FITC, APC, APC-H7, and PerCP-Cy5.5. The labeled cells were analyzed on a LSR II flow cytometer (Becton Dickinson, Lakes, NJ).

## 3 Results

### 3.1 High incidence of the CD22ΔE12 genetic defect in B-lineage lymphoid malignancies

**Fig. 1:**
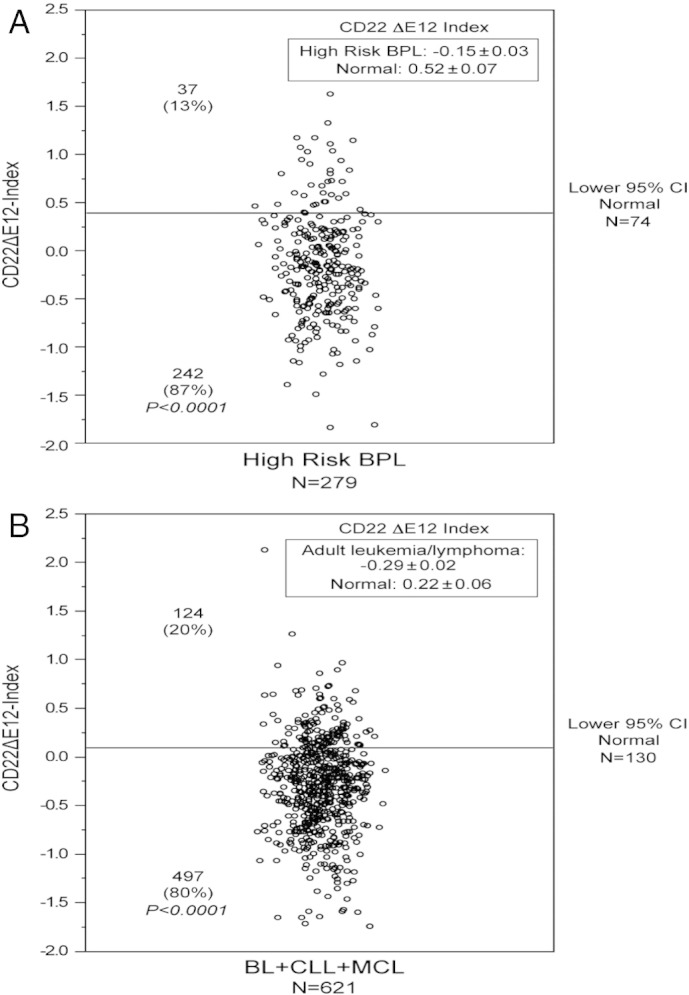
Incidence of CD22ΔE12 in pediatric and adult B-lineage lymphoid malignancies. BLAT analysis on CD22 probe sequences for probeset 217422_s_at (covering exons 10–14) deposited in the Affymetrix NetAffx™ Analysis Center were mapped onto specific CD22 exons and visualized using the UCSC genome browser (http://genome.ucsc.edu/cgi-bin/hgBlat?command=start). An Exon 12 Index was calculated by subtracting the median centered expression values of the 7 probes for Exons 10–11 and Exons 13–14 from the median centered expression values of the 3 Exon 12 probes. [A] Depicted is the dot plot of the sample level CD22ΔE12-index values in primary leukemia/lymphoma samples from 621 adult patients with Burkitt 's lymphoma, CLL or mantle cell lymphoma/leukemia. 497 samples (80%) leukemic samples had lower CD22ΔE12-index values that were lower than the 95% lower confidence interval for the CD22ΔE12-index values for the 130 normal B-cell samples (95% CI = 0.094–0.349) (P < 0.0001). The average CD22ΔE12 index values were − 0.29 ± 0.02 for the leukemia/lymphoma samples and 0.22 ± 0.01 for the normal B-cell samples. [B] Depicted is the dot plot of the sample level CD22ΔE12-index values in primary leukemia samples from 279 pediatric BPL patients and 74 normal samples (95% CI = 0.394–0.655). 242 samples (87%) leukemic samples had lower CD22ΔE12-index values that were lower than the 95% lower confidence interval for the CD22ΔE12-index values for the 74 normal bone marrow samples (P < 0.0001). The average CD22ΔE12 index values were − 0.15 ± 0.03 for the leukemia samples and 0.52 ± 0.07 for the normal bone marrow samples. BL: Burkitt's lymphoma, CLL: chronic lymphocytic leukemia, DLBCL: diffuse large B-cell lymphoma, FL: follicular lymphoma, MCL: mantle cell leukemia/lymphoma.

A CD22ΔE12-index was calculated by subtracting the median centered expression values of the 7 probes for CD22 Exons 10–11 and CD22 Exons 13–14 in the 217422_s_at probeset from the median centered expression values of the 3 CD22 Exon 12 probes. We found highly significant reductions in Exon 12 index for primary leukemia cells in newly diagnosed high-risk pediatric B-precursor ALL patients. 242 of 279 (87%) leukemic samples had lower CD22ΔE12-index values that were lower than the 95% lower confidence interval for the CD22ΔE12-index values for the normal hematopoietic cells in 74 non-leukemic control bone marrow samples (P < 0.0001). The average CD22ΔE12 index values were − 0.15 ± 0.03 for the leukemia samples and 0.52 ± 0.07 for the normal bone marrow samples (Fig. 1A). Likewise, adult B-lineage lymphoid malignancies were characterized by a high incidence of the CD22ΔE12 genetic defect. Leukemia/lymphoma cells from 497 of 621 patients with B-lineage lymphoid malignancies (80%) had lower CD22ΔE12-index values that were lower than the 95% lower confidence interval for the CD22ΔE12-index values for the 130 normal B-cell samples (95% CI = 0.094–0.349) (P < 0.0001). The average CD22ΔE12 index values were − 0.29 ± 0.02 for the leukemia/lymphoma samples and 0.22 ± 0.01 for the normal B-cell samples (Fig. 1B).

### 3.2 Signature phosphoproteome of CD22ΔE12 transgenic mouse BPL cells

**Fig. 2:**
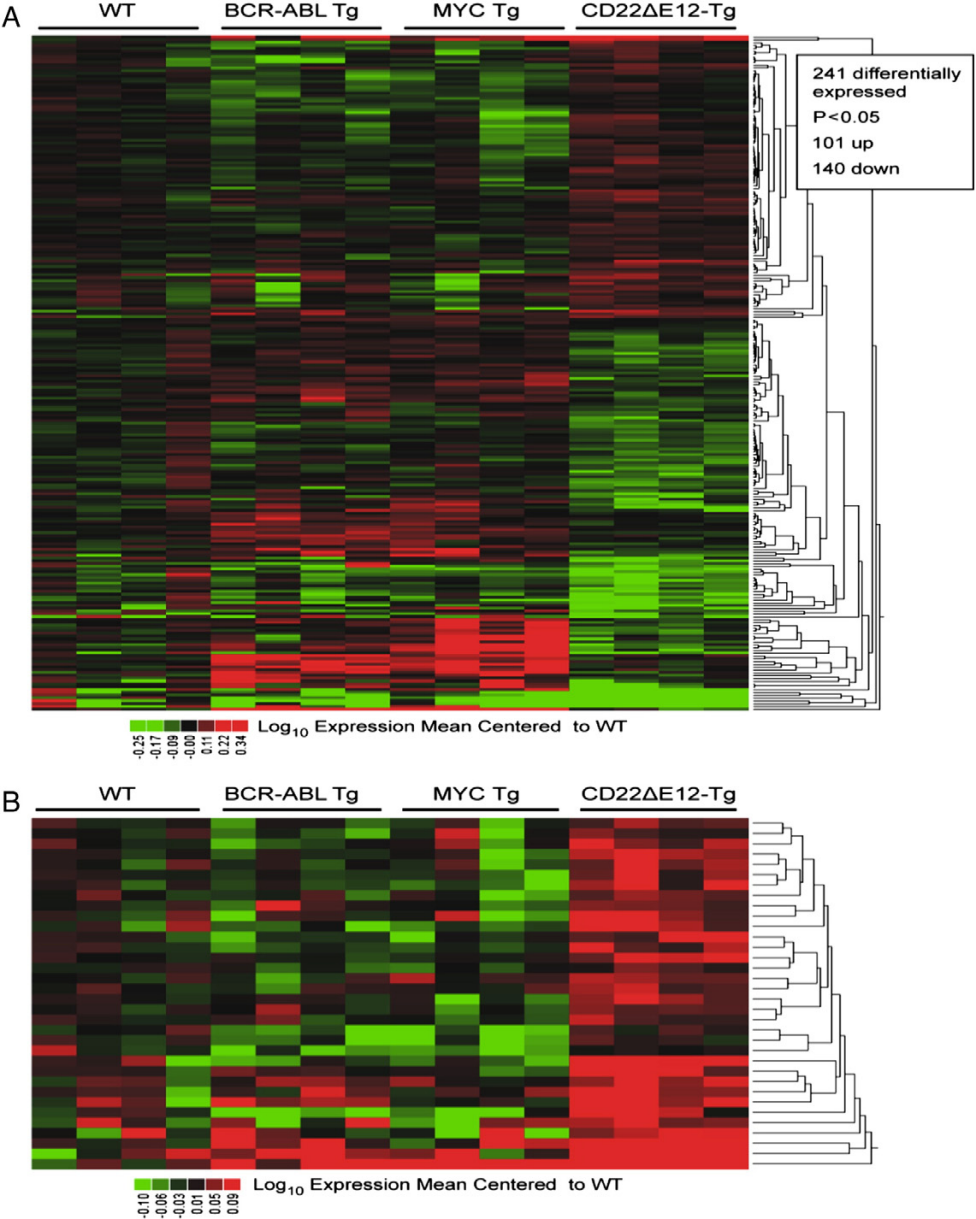
Signature phosphoproteome of CD22ΔE12 transgenic mouse BPL cells. The phosphoprotein profiles of CD22ΔE12-Tg BPL cells were compared side-by-side with those of Eμ-MYC Tg BPL cells, BCR-ABL Tg BPL cells as well as WT splenocytes from healthy C57BL/6 mice by using the Phospho Explorer Antibody Array platform (Full Moon BioSystems, Inc., Sunnyvale, CA), as described in Methods section. Data were normalized utilizing the median intensity values for the 1318 antibodies on each array. The normalized data were log_10_ transformed and mean centered to the WT C57BL/6 splenocyte samples (N = 4, 2 technical replicates for each of 2 samples). [A] Depicted is a one-way hierarchical cluster figure that illustrates phosphoprotein expression differences between BPL cells from CD22ΔE12-Tg mice and normal splenocytes from healthy WT mice, BPL cells from BCR-ABL Tg mice or BPL cells from Eμ-MYC Tg mice. 241 proteins were differentially expressed in CD22ΔE12-Tg BPL cells (P < 0.005): 101 proteins were upregulated and 140 proteins were downregulated. Heat map depicts up and down regulated protein expression levels ranging from red to green respectively. [B] Depicted are the most significantly overexpressed 32 phosphoproteins (represented by 34 specific antibodies used as probes in CD22ΔE12-Tg mice). Expression values were mean centered to WT samples and clustered according to average distance metric. The results of the Student's T-tests (Unequal variance correction, Microsoft Excel) comparing mean-centered, log_10_ transformed protein expression levels in BPL cells from CD22ΔE12-Tg mice vs. splenocytes from WT mice and BPL cells from BCR-ABL Tg or MYC Tg mice are detailed in supplemental Table S1.

The phosphoprotein profiles of CD22ΔE12-Tg BPL cells were compared side-by-side with those of Eμ-MYC Tg BPL cells, BCR-ABL Tg BPL cells as well as WT splenocytes from healthy C57BL/6 mice by using the Phospho Explorer Antibody Array platform, as described in Methods. Data were normalized utilizing the median intensity values for the 1318 antibodies on each array. The normalized data were log_10_ transformed and mean centered to the WT C57BL/6 splenocyte samples (N = 4, 2 technical replicates for each of 2 samples). 241 proteins (101 upregulated and 140 downregulated) were differentially expressed in CD22ΔE12-Tg BPL cells (P < 0.005) (Fig. 2A). The most significantly overexpressed 32 proteins (represented by 34 specific antibodies) included several proteins known for their anti-apoptotic function (e.g. mTOR, p70S6, AKT, NFκB), transcription factors implicated in oncogenesis (e.g. ATF2), and serine kinase signaling pathway proteins (e.g. MAPK, PKC, PKD) (Fig. 2B, Table S1).

### 3.3 CD22ΔE12-driven gene expression cassette characterizes primary human leukemia cells

**Fig. 3:**
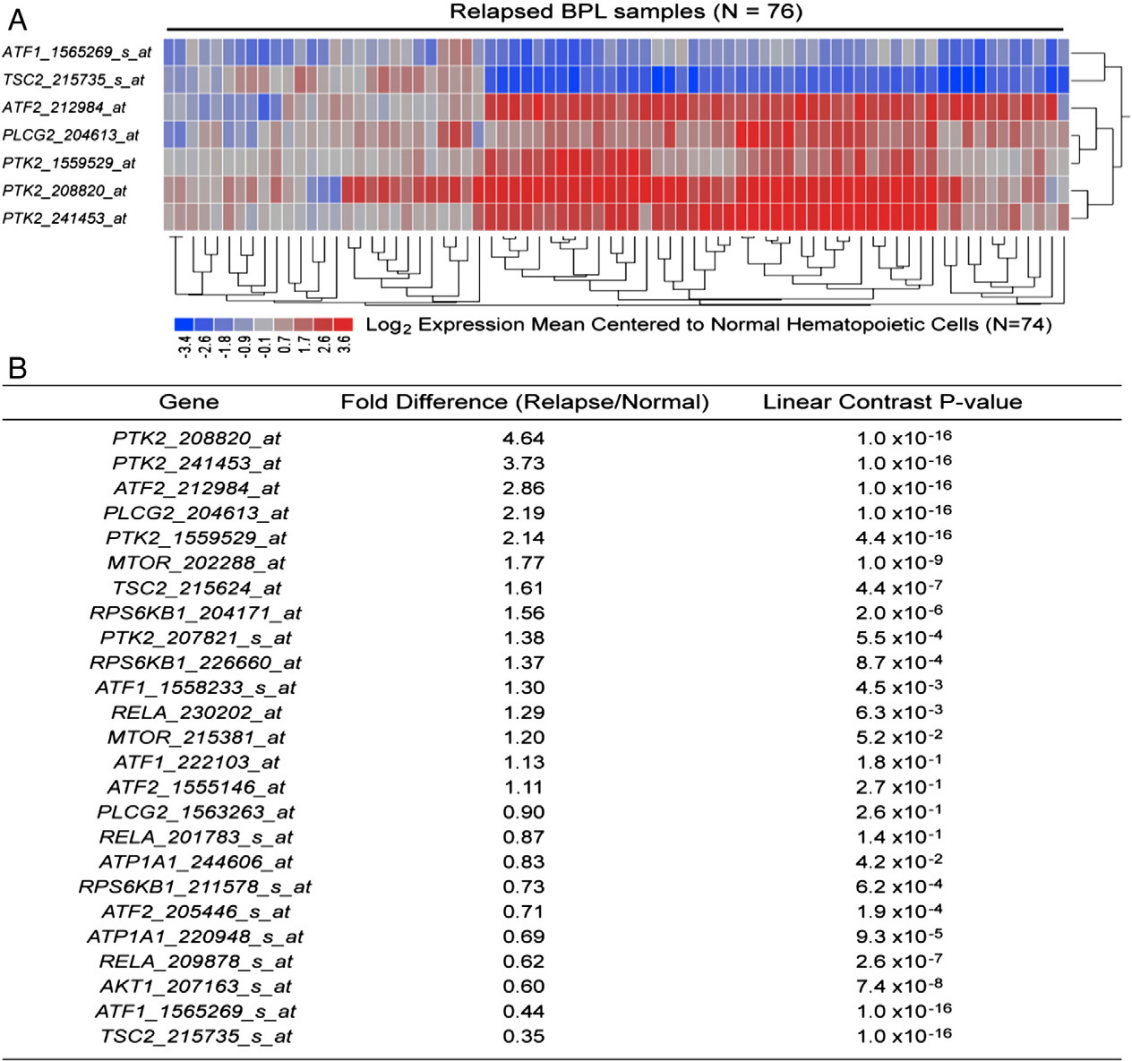
Upregulated expression of the CD22ΔE12-driven gene expression cassette in human leukemia cells from pediatric patients with relapsed BPL. The 10-gene mouse CD22ΔE12-Tg gene expression cassette was represented by 25 probesets on the human U133 plus 2.0 array. The RMA-normalized gene expression values for leukemia cells from 76 BPL patients in relapse were log_2_ transformed and mean-centered to the average value for the normal hematopoietic cells (N = 74). [A] The gene expression values were clustered according to average distance metric. Heat map depicts up and down regulated transcripts ranging from red to blue respectively for expression values mean centered to normal hematopoietic cells in non-leukemic samples. [B] To determine the differential expression of each leading edge gene of the CD22ΔE12 transcriptome in relapsed BPL cells, linear contrasts were performed for the RMA normalized values (P < 0.05 deemed significant). Depicted are the mean fold difference and linear contrast P-values relative to normal samples for the comparisons ordered according to effect size. Mixed Model ANOVA demonstrated significant increases in the multivariate means for the 25 probesets for Normal vs. Relapsed BPL (Diagnostic group effect, F_1,148_ = 11.7, P = 0.0008). Five transcripts representing 3 genes displayed greater than 2 fold increases in relapsed BPL cases (PTK2_208820_at, PTK2_241453_at, ATF2_212984_at, PLCG2_204613_at, PTK2_1559529_at) and 12 transcripts representing 8 genes (ATF1, ATF2, MTOR, PLCG2, PTK2, RELA, RPS6KB1 and TSC2) were upregulated with P < 0.05.

**Fig. 4:**
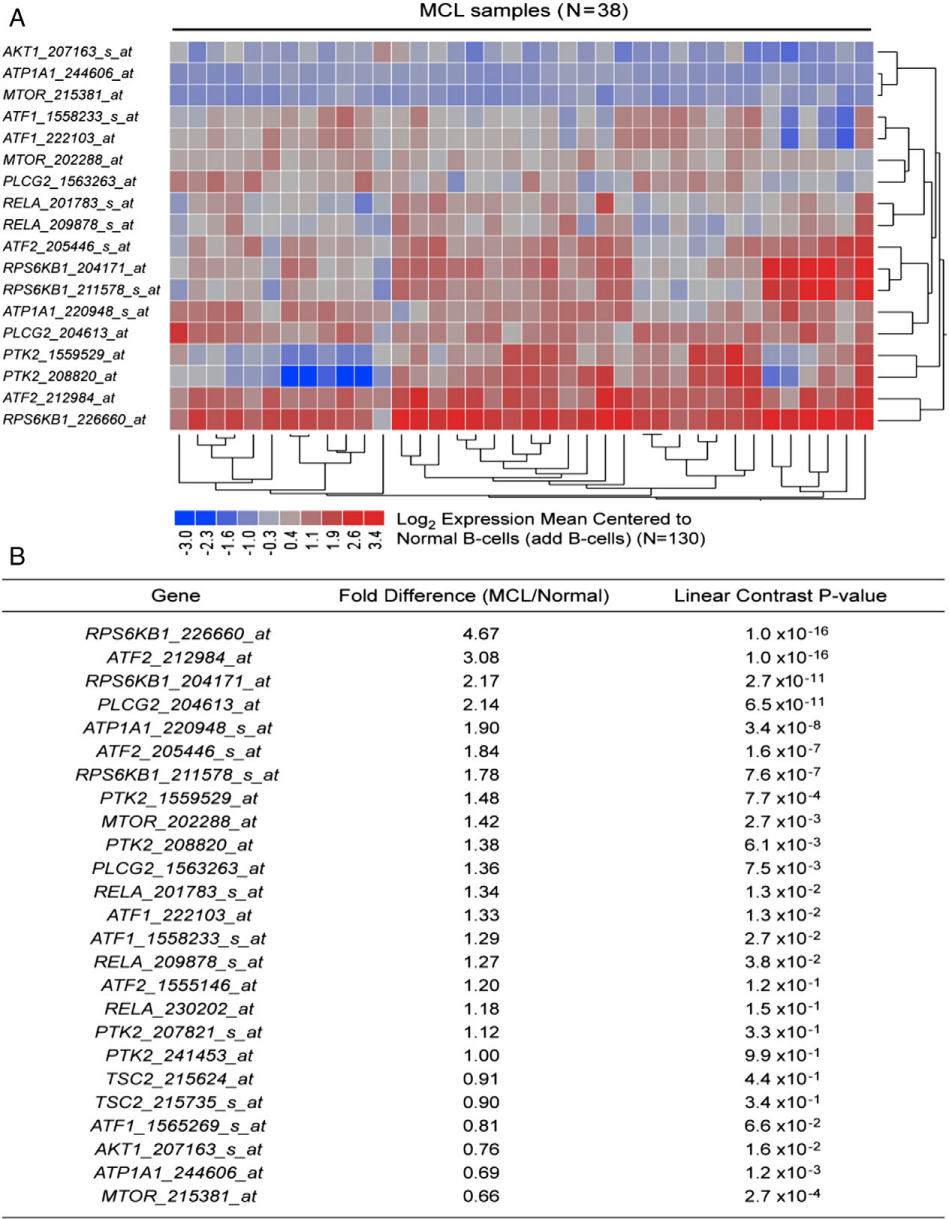
Upregulated expression of the CD22ΔE12-driven gene expression cassette in human leukemia cells from adult patients with MCL. The 10-gene mouse CD22ΔE12-Tg gene expression cassette was represented by 25 probesets on the human U133 plus 2.0 array. The RMA-normalized gene expression values for leukemia cells from 38 patients with MCL were log_2_ transformed and mean-centered to the average value for the normal B-cells (N = 130). [A] The gene expression values were clustered according to average distance metric. Heat map depicts up and down regulated transcripts ranging from red to blue respectively for expression values mean centered to normal B-cells in non-leukemic samples. [B] To determine the differential expression of each leading edge gene of the CD22ΔE12 transcriptome in MCL cells, linear contrasts were performed for the RMA normalized values (P < 0.05 deemed significant). Mixed model ANOVA demonstrated significant increases in the multivariate means for the 25 probesets for Normal vs. MCL (diagnostic group effect F_1,166_ = 13.1, P = 0.0004). Depicted are the mean fold difference and linear contrast P-values relative to normal samples for the comparisons ordered according to effect size. Four transcripts representing 3 genes were significantly up regulated greater than 2-fold (RPS6KB1, ATF2, PLCG2) and 15 transcripts representing 8 genes were up regulated with P-values < 0.05 (ATF1, ATF2, ATP1A1, MTOR, PLCG2, PTK2, RELA and RPS6KB1).

We examined the gene expression profiles of the CD22ΔE12-Tg, MYC-Tag and BCR-ABL-Tag BPL cells vs. wild type normal splenocytes using the Mouse Gene 1.0 ST oligonucleotide array platform and performed linear contrasts to determine if the expression levels of any 32 genes (represented by 43 probesets) that encode differentially upregulated phosphoproteins in CD22ΔE12-Tg BPL cells were also differentially upregulated in CD22ΔE12-Tg BPL cells. Transcript levels were significantly higher in CD22ΔE12-Tg BPL cells for 10 of these 32 genes represented by 25 probesets (Fig. S1). Notably, this 10-gene signature transcriptome was also upregulated in Uckun et al. (2010) BPL cells from 76 relapsed pediatric BPL patients (GSE28460 and GSE18497) vs. normal hematopoietic cells (Fig. 3) (Ma et al., 2012), leukemic B-cells from 578 adult CLL patients (GSE13159 and GSE39671) vs. non-leukemic B-cells (Fig. S2), and (Uckun et al., 1987) neoplastic cells from 38 MCL patients (GSE36000) vs. normal B-cells (Fig. 4). Mixed Model ANOVA demonstrated significant increases in the multivariate means for the 25 probesets for relapsed BPL (diagnostic group effect, F_1,148_ = 11.7, P = 0.0008), CLL (F_1,706_ = 15.7, P = 0.00008) as well as MCL (F_1,166_ = 13.1, P = 0.0004). Four transcripts representing 3 genes were significantly upregulated across all 3 patient groups of leukemia patients (ATF2_212984_at, PLCG2_204613_at, RPS6KB1_204171_at, RPS6KB1_226660_at). Significant negative correlations between CD22ΔE12 index and expression of each of the 4 probesets (PLCG2_204613_at, r = − 0.35, F_1,894_ = 124, P < 0.0001; RPS6KB1_204171_at, r = − 0.22, F_1,894_ = 47, P < 0.0001; RPS6KB1_226660_at, r = − 0.18, F_1,894_ = 28, P < 0.0001; ATF2_212984_at, r = − 0.14, F_1,894_ = 17, P < 0.0001) were observed suggesting that a loss of expression of CD22 Exon 12 results in the up regulation of these most differentially upregulated CD22ΔE12-driven signature genes (Fig. S3).

### 3.4 Effect of CD22ΔE12–14 vs. CD22^WT^ on clonogenicity and self-renewal rate of leukemia cell lines

**Fig. 5:**
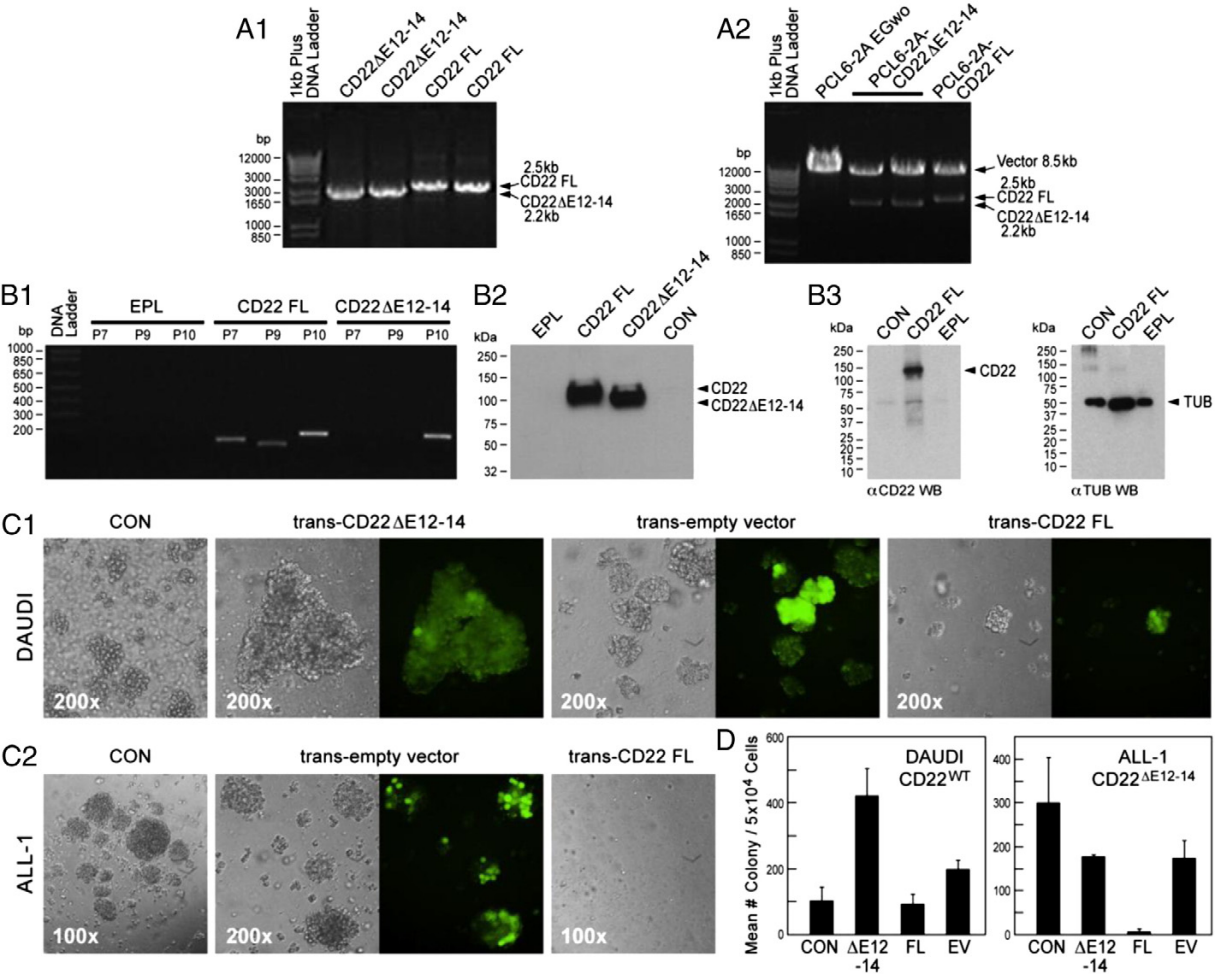
Effect of CD22ΔE12 vs. CD22^WT^ on clonogenicity and self-renewal rate of ALL cells. [A1] Depicted is a representative 1% agarose gel showing the PCR products of FL CD22 and CD22ΔE12–14. [A.2] Subcloning of the CD22 FL or CD22ΔE12–14 genes into the lentiviral pCL6-2AEGwo vector through NheI and XhoI restriction sites was confirmed through restriction enzyme digestion and DNA sequencing. Depicted is a representative 1% agarose gel showing that the generated lentiviral constructs have the correct size FL CD22 (2.5-kb) or CD22ΔE12–14 (2.2-kb) inserts. [B.1] In order to confirm that the lentiviral vectors can be used to achieve expression of FL and truncated CD22 in human cells, 293T cells were transfected with lentiviral constructs for FL CD22, CD22ΔE12–14, as well as pCL6-2AEGwo lentiviral vector without any subcloned CD22. 48 h post transfection cells were examined for FL CD22 and CD22ΔE12–14 mRNA by RT-PCR using the P7, P9, and P10 primer pairs. The P7 primer set was used to amplify a 182-bp region (c.2180–c.2361) of the CD22 cDNA extending from Exon 11 to Exon 13 and spanning the entire Exon 12. The P9 primer set was used amplify a 160-bp region (c.2304–c.2463) of the CD22 cDNA extending from Exon 12 to Exon 14 and spanning the entire Exon 13. The P10 primer set was used to amplify a 213-bp region (c.433–c.645) of Exon 4 of the CD22 cDNA present in both wildtype CD22 and CD22ΔE12–14 mRNA species. As expected all primer sets yielded PCR products in cells transfected with the FL CD22 vector and only the P10 primer set yielded a PCR product in cells transfected with the CD22ΔE12–14 vector. [B.2 and B3] The increased expression levels of the full-length and truncated proteins in transduced 293-T cells (depicted in B.2) and ALL-1 cells (depicted in B.3) were confirmed to be similar by Western blot analysis done at 96 h post-transduction. [C & D] ALL cell lines DAUDI (Burkitt's leukemia/B-ALL) (panels C1 & C.2) and ALL1 (BCR-ABL^+^ B-precursor ALL) (panel C3) were transduced (trans) with wildtype and mutant human CD22 genes using the pCL6-2AEGwo lentiviral vector and then assayed for colony formation in semi-solid methylcellulose cultures without additional stroma support or cytokines. Colony formation was examined using an inverted Nikon Eclipse TS100 microscope with an Epifluorescence attachment. Images were taken using a Digital Sight DS-2MBW Nikon camera (System magnification: 100× or 200 × as indicated). Green fluorescence of colonies resulting from GFP expression confirms successful transduction of the cell lines. [D] Depicted are bar graphs comparing the mean colony numbers in cultures of untransduced and transduced cells.

CD22ΔE12 is a mis-spliced mRNA resulting from an intronic mutation that skips exon 12. Translation of CD22ΔE12 sequence into protein results in a truncating frame shift starting at residue 736. CD22ΔE12 protein is unable to transmit apoptotic signals, as most of the intracellular domain encoded by Exons 12–14 is lost, including the key regulatory signal transduction elements and all of the cytoplasmic tyrosine residues (Uckun et al., 2010). In an effort to evaluate the effects of expression of a truncated CD22 protein on the clonogenicity and self-renewal rate of leukemia and lymphoma cell lines, we cloned wildtype full-length (FL) human CD22 cDNA and a truncated human CD22 cDNA that lacks exons 12–14 (CD22ΔE12–14) into the pCL6-2AEGwo lentiviral vector containing a 2A-like peptide (Figs. S4, 5A1, A2 & B1). The expression levels of the full-length and truncated proteins were confirmed to be similar by Western blot analysis (Fig. 5B2 & B3). Expression of GFP-tagged full length and truncated CD22 proteins in transduced leukemia cells was further confirmed by fluorescence microscopy (Fig. 5C). In two independent experiments, lentiviral-based expression of truncated CD22ΔE12–14 protein (but not FL CD22) in CD22ΔE12-negative Burkitt's leukemia/lymphoma cell line DAUDI resulted in a marked increase in the size of day 7 colonies on consistent with increased self-renewal as well as increase of its in vitro clonogenicity, as measured by increased number (per 100,000 cells) of colonies formed in semisolid methylcellulose cultures (421 ± 83 vs. 104 ± 40, linear contrast P-value = 0.0006) (Fig. 5C & D). Conversely, in both experiments, the lentiviral-based overexpression of FL CD22 in CD22ΔE12-positive ALL-1 cell line virtually abrogated its clonogenic growth (8 ± 5 vs. 301 ± 103, linear contrast P-value = 0.0025). The empty vector without an insert did not consistently affect the clonogenicity or self-renewal of either cell line (Fig. 5C & D). These findings support our hypothesis that expression of a truncated human CD22 that lacks the signal transduction elements encoded by Exons 12–14, as it occurs in CD22ΔE12^+^ ALL, would confer B-lineage leukemia and lymphoma cells with a selective growth advantage.

### 3.5 CD22ΔE12 as a molecular target for RNAi therapy in human B-lineage lymphoid malignancies

**Fig. 6:**
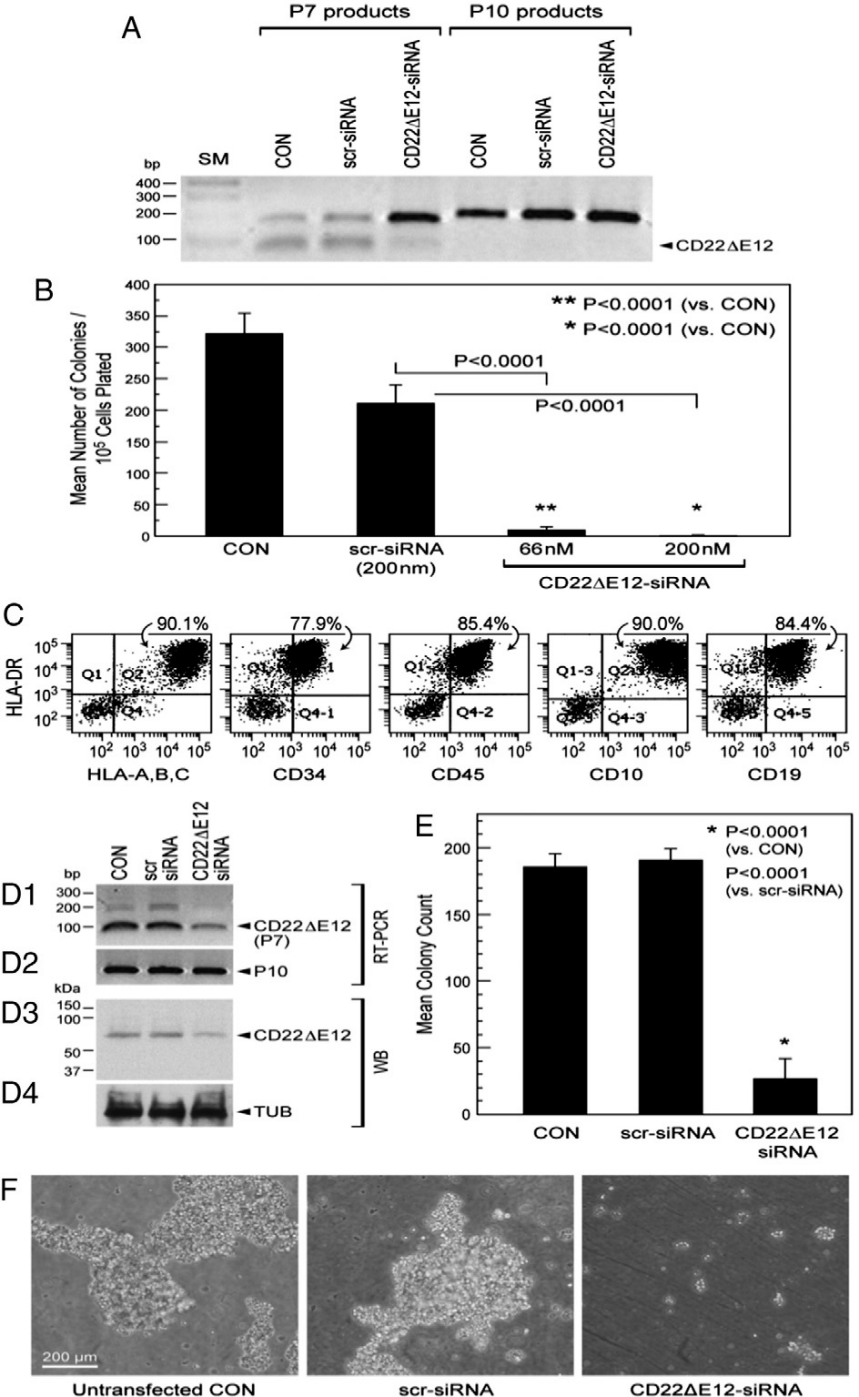
CD22ΔE12 siRNA induced selective depletion of aberrant CD22ΔE12 mRNA species. [A] Cells were transfected with a Nucleofector II device (Amaxa GmbH; protocol VCA-1002/C005) with either CD22ΔE12-siRNA or scr-siRNA. Controls included untreated cells. Depicted is the image of a 1% agarose gel showing the RT-PCR products obtained from the RNA samples of CD22ΔE12-siRNA transfected ALL-1 cells using the P7 primer pair to amplify a 182-bp region (c.2180–c.2361) of the CD22 cDNA extending from Exon 11 to Exon 13 and spanning the entire Exon 12. Deletion of Exon 12 results in a smaller CD22ΔE12-specific PCR product of 63-bp size with this primer set migrating slightly below the 100-bp size marker. The P10 primer set was used as a positive control of RNA integrity to amplify a 213-bp region (c.433–c.645) of the CD22 cDNA present in both wildtype CD22 and CD22ΔE12 mRNA species. CD22ΔE12-siRNA but not scr-siRNA caused depletion of the CD22ΔE12 mRNA. [B] Depicted are the mean numbers of colonies per 100,000 ALL-1 cells plated after transfection with CD22ΔE12-siRNA (66 nM or 200 nM) or scr-siRNA (200 nM). Controls included untreated cells (CON). [C] Depicted are representative FACS-correlated two-parameter displays of BPL xenograft cells isolated from spleens of NOD/SCID mice that developed overt leukemia after inoculation with primary ALL cells from BPL patients. Cells were stained by direct immunofluorescence for human lymphoid differentiation antigens CD10, CD19, CD34, CD45, HLA-DR/DP/DQ, and HLA-A,B,C. The labeled cells were analyzed on a LSR II flow cytometer. Xenograft cells co-expressed CD45, HLA-DR/DP/DQ and HLA-A,B,C antigens and had an immature B-cell precursor immunophenotype characterized by co-expression of B-lineage progenitor antigens CD10/CALLA, CD19, and CD34. [D] Depicted is the image of a 1% agarose gel showing the RT-PCR product obtained from the RNA sample of CD22ΔE12-siRNA (50 nM) transfected xenograft cells using the P7 primer pair. The P10 primer set was used as a positive control of RNA integrity. Whole cell lysates of xenograft cells were subjected to CD22 and alpha-tubulin (TUB) Western blot analysis. The positions of truncated mutant CD22ΔE12 protein and TUB are indicated with arrowheads. BPL xenograft cells expressed a CD22ΔE12-associated truncated CD22 protein instead of the 130/140-kDa intact CD22 protein. Transfection with CD22ΔE12-siRNA, but not scrambled (scr)-siRNA, caused selective depletion of CD22ΔE12-mRNA as well as CD22ΔE12-protein in aggressive BPL xenograft cells. [E] The bar graphs depict the mean ± SE values for blast colonies per 100,000 cells plated for all ALL xenograft clones (N = 3). While untransfected xenograft cells formed 186 ± 9 colonies, CD22ΔE12-siRNA transfected xenograft cells formed only 27 ± 14 colonies (P < 0.0001). By comparison, scr-siRNA transfected xenograft cells formed 191 ± 24 colonies, which was not significantly different from the colony numbers obtained in cultures of untransfected cells (P = 0.8) but significantly higher than those in cultures of CD22ΔE12-siRNA transfected xenograft cells (P < 0.0001). [F] Colony formation was examined using an inverted Nikon Eclipse TS100 microscope. Images were taken using a Digital Sight DS-2MBW Nikon camera. Depicted are images of blast colonies demonstrating that CD22ΔE12-siRNA transfection results in reduction of the number as well as size of blast colonies.

We first used RT-PCR to examine the effects of transfection with CD22ΔE12-specific siRNA on CD22ΔE12 mRNA expression levels in CD22ΔE12^+^ BPL cell line ALL-1 with homozygous intronic CD22 gene mutations at Rs10413526 (C>G) and Rs4805120 (A>G). CD22ΔE12-siRNA (but not scrambled/scr-siRNA) caused depletion of the truncated CD22ΔE12-mRNA (Fig. 6A). CD22ΔE12-depletion in transfected ALL-1 was associated with abrogation of in vitro proliferation and clonogenicity, as reflected by a drastic decrease in the number and cellular content of colonies formed in semi-solid methylcellulose cultures (Fig. 6B). We next used aggressive BPL xenograft clones (N = 3) isolated from spleens of NOD/SCID mice that had developed overt leukemia with massive splenomegaly following inoculation with primary leukemia cells from the respective BPL patients to further evaluate the suitability of CD22ΔE12 as a molecular target for RNAi. Immunophenotyping by multiparameter flow cytometry confirmed the co-expression of multiple BPL-associated human lymphoid differentiation antigens, including high levels of CD10, CD19, and CD34 (Fig. 6C) that have been reported as markers of putative leukemic stem cells in BPL (Uckun et al., 2013). Transfection with CD22ΔE12-siRNA, but not scr-siRNA, caused selective (albeit partial) depletion of CD22ΔE12-mRNA as well as CD22ΔE12-protein in aggressive BPL xenograft cells (Fig. 6D). This siRNA-mediated CD22ΔE12-knockdown was associated with a marked inhibition of their clonogenic growth in vitro (Fig. 6E). The mean number of blast colonies per 100,000 (Uckun et al., 1987) BPL xenograft cells plated were 186 ± 18 for untransfected control cells, 191 ± 24 for cells transfected with scr-siRNA (60 nM), and 27 ± 14 for cells transfected with CD22ΔE12-siRNA (60 nM) (P < 0.0001 vs. scr-siRNA, P < 0.0001 vs. CON). Both the proliferative activity and clonogenicity were inhibited, as reflected by the substantially smaller number and size of the blast colonies (Fig. 6F1–F3).

### 3.6 Targeting leukemia cells with CD22ΔE12-siRNA nanoparticles

**Fig. 7:**
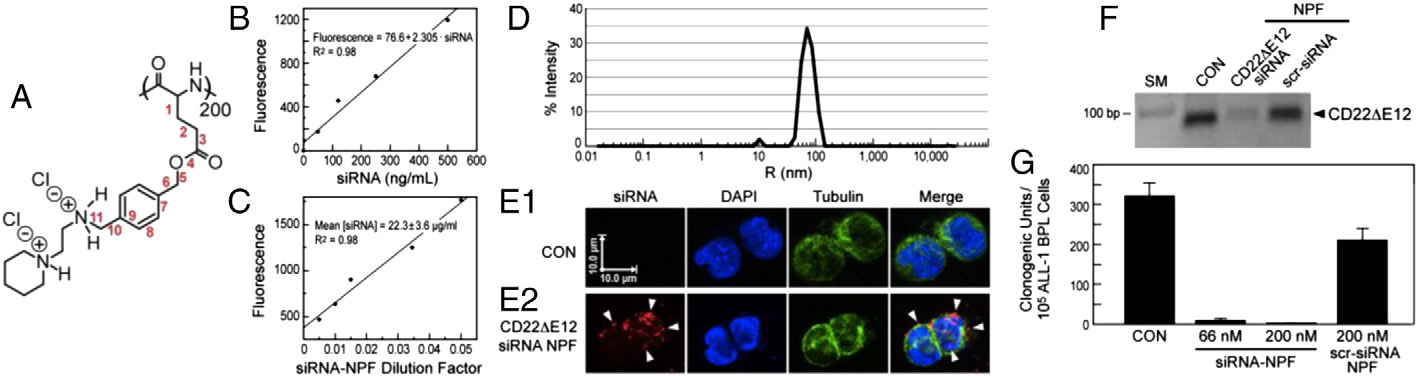
PVBLG-8-based nanoformulation (NF) of CD22ΔE12-siRNA. [A] Chemical structure of the 200-mer polymer of PVBLG-8 that we used to complex siRNA as recently published (Gabrielson et al., 2012, Lu et al., 2011, Yin et al., 2013a). The MW for each repeating unit is 430. [B & C] Determination of the siRNA content using the standard Quant-iT RiboGreen RNA assay (Invitrogen) and a Synergy HT Biotek fluorescence microplate reader to measure the siRNA content. The standard curve is depicted in B. [D] DLS curve of the complex confirming that we achieved a nanoscale formulation. [E1 & E2] We used 5 ' Cy3-labeled CD22ΔE12-siRNA to prepare a CD22ΔE12-siRNA “nanoparticle” formulation (NPF) for cellular uptake and trafficking experiments using confocal imaging, as described in Fig. 4. After incubation with the PVBLG-8 based formulation of Cy3-labeled siRNA for 6 h, the internalized Cy3-labeled CD22ΔE12-siRNA was detected and localized using the TRITC filter sets. Arrowheads in DAPI/tubulin/siRNA merge images point to location of Cy3-labeled siRNA molecules inside the DAPI-stained (blue) nucleus and -containing (green) perinuclear cytoplasm. [F] RT-PCR was performed with the P7 primer pair as in Fig. 2 (Ma et al., 2012). [G] 100,000 ALL-1 cells were plated in duplicate dishes in methylcellulose cultures containing no NPF (CON), 66 nM or 200 nM of the CD22ΔE12-siRNA NPF, or 200 nM of the scrambled (scr)-siRNA NPF. Colonies were counted after 7 days. Depicted are bar graphs showing the mean clonogenic units per 100,000 cells plated using the cumulative data from 3 experiments. The mean ± SE values were 322.0 ± 32.1 for CON, 211.8 ± 28.3 for 200 nM scr-siRNA NPF, 10.8 ± 4.3 for 66 nM and 0.2 ± 0.2 for 200 nM CD22ΔE12-siRNA NPF.

We recently reported a cell-penetrating cationic helical polypeptide (termed PVBLG-8) that displays distinguished membrane disruption and endosomal escape properties (Gabrielson et al., 2012, Lu et al., 2011). PVBLG-8 was identified as the top-performing peptide for nucleoside delivery and preparation of nanoscale siRNA formulations (Yin et al., 2013a). (Fig. S5). PVBLG-8 was synthesized via controlled ring opening polymerization of γ-(4-vinylbenzyl)-l-glutamate NCA (VB-l-Glu-NCA), as initiated by hexamethyldisilazane (HMDS) followed by multi-step side-chain modification (Fig. S5A) (Gabrielson et al., 2012, Lu et al., 2011, Yin et al., 2013a, Zhang et al., 2011). In an attempt to further strengthen the electrostatic interactions of the cationic PVBLG-8 with the CD22ΔE12-siRNA and promote the CD22ΔE12-siRNA encapsulation in the complex nanostructure, we also included another anionic polypeptide, PAOBLG-MPA (Zhang et al., 2011) in the formulation at a PVBLG-8:PAOBLG-MPA:CD22ΔE12-siRNA weight ratio of 10:2:1 (Fig. S5B). By using this formulation strategy, we have successfully complexed CD22ΔE12-siRNA with a 200-mer polymer of PVBLG-8 to prepare a nanoscale formulation of CD22ΔE12-siRNA (Fig. 7D). This unique nanoparticle formulation effectively delivered Cy3-labeled CD22ΔE12-siRNA into the cytosol of ALL-1 cells (Fig. 7E), caused marked CD22ΔE12 depletion, as determined by RT-PCR with the P7 primer pair (Fig. 7F) and inhibited their clonogenic growth in vitro (Fig. 7G). At both concentrations used, its inhibition of the ALL-1 clonogenicity (96.6% at 66 nM and 99.94% at 200 nM) was much more pronounced than the non-specific effects of the scr-siRNA formulation (34.2% at 200 nM) (Fig. 7G). The linear contrast P-values for CD22ΔE12-siRNA NF vs. CON + scr-NF were 9 × 10^− 10^ for 66 nM and 4 × 10^− 10^ for 200 nM.

## 4 Discussion

The major challenge in the treatment of BPL is to cure patients who have relapsed despite intensive frontline chemotherapy. There is an urgent and unmet need to identify new drug candidates capable of destroying chemotherapy-resistant leukemic B-cell precursors (BCP). Our recent findings have implicated CD22ΔE12 arising from a splicing defect associated with homozygous intronic mutations as a previously undescribed pathogenic mechanism in human BPL (Uckun et al., 2010). CD22ΔE12 is the first reported genetic defect implicating a B-cell co-receptor in a human lymphoid malignancy and linking homozygous mutations of the CD22 gene to a human disease and also the first genetic defect implicating intronic mutations in the pathogenesis of leukemia. The abnormal CD22ΔE12 protein is encoded by a profoundly aberrant mRNA arising from a splicing defect that causes the deletion of exon 12 (c.2208–c.2327) (CD22ΔE12) and results in a truncating frame shift mutation. Our overarching objective in this project was to design an innovative and effective strategy that would utilize the CD22ΔE12 genetic lesion as a molecular target to gain a therapeutic advantage against chemotherapy-resistant aggressive BPL. Specifically, we sought to validate CD22ΔE12 as an effective molecular target for RNA interference (RNAi) therapy in BPL. Transfection of leukemia cells with CD22ΔE12-specific siRNA caused effective depletion of the truncated CD22ΔE12-mRNA. CD22ΔE12-depletion was a drastic inhibition of the proliferative activity and clonogenicity of leukemia cells, including aggressive leukemia xenograft clones. These results provided unprecedented evidence that the CD22ΔE12 genetic defect can be leveraged as a molecular target for RNAi therapy against B-lineage lymphoid malignancies.

Nanoparticles represent particularly attractive delivery systems for small interfering RNA (siRNA) and may provide the foundation for rational design and formulation of RNAi-triggering nanomedicines. We recently developed a platform for the facile generation of cationic helical polypeptides (termed PVBLG), which was realized by maintaining a separation distance of 11 σ-bonds between the peptide backbone and the side chain charge (Gabrielson et al., 2012, Lu et al., 2011). Based on this strategy, a library of cationic polypeptides with purposely designed, stable helical structures were prepared. Like cell-penetrating peptides found in nature, these cationic helical polypeptides displayed distinguished membrane disruption/destabilization properties. Subtle changes of side chains allowed the generation of polypeptides with appropriate DNA binding strength as well as endosomal escape capacity. PVBLG-8 was identified as the top-performing peptide for gene transfection (Gabrielson et al., 2012, Lu et al., 2011, Yin et al., 2013a). We developed it further as a nanoscale siRNA delivery platform and have recently provided the first preclinical proof of principle that this helical polypeptide is uniquely suited for preparation of nanoscale siRNA formulations (Yin et al., 2013a).

In the present study, we used a polymer of PVBLG-8 to prepare a nanoscale formulation of CD22ΔE12-siRNA. PVBLG-8 with its long back-bone length and high charge density provided the desired affinity for the oppositively charged siRNA molecules, whereas the helical secondary structure of PVBLG-8 was designed to facilitate the delivery of siRNA into leukemia cells and their escape from the endosomal/lysosomal compartments. The nanoparticle formulation was highly effective in knocking down the expression of CD22ΔE12 and killing clonogenic leukemia cells. Further development and optimization of this RNAi therapeutic candidate targeting CD22ΔE12 may facilitate a paradigm shift in therapy of aggressive or chemotherapy-resistant B-lineage lymphoid malignancies.

A more rapid development of nanoscale RNAi therapeutics has been hampered by the limited knowledge about the identity of the critical driver lesions in specific types of cancer, safety concerns about certain formulations, and a disappointingly poor delivery of the siRNA into target cancer cells (Guo & Huang, 2011). Only a limited number of polymeric materials have been tested in clinical trials for their capability of delivering siRNA into human cancer cells. Examples include the linear cyclodextrin polycations developed by Mark Davis at Caltech/Calando Pharmaceuticals (Davis et al., 2010) and the siG12D LODER (a proprietary biodegradable polymeric matrix) developed by Silenseed Science and Technology (Burnett et al., 2011). There is an urgent and unmet need to identify novel materials and delivery systems capable of safely and efficiently delivering siRNA to molecular targets not amenable to other drug-delivery approaches. The described siRNA delivery platform is based on a class of rationally designed cell-penetrating cationic helical polypeptides (Gabrielson et al., 2012, Lu et al., 2011, Yin et al., 2013a, Zhang et al., 2011). These polypeptide-based novel nanomaterials exhibit unprecedented thermodynamic and physicochemical stability (Gabrielson et al., 2012, Lu et al., 2011, Yin et al., 2013a, Zhang et al., 2011) and unique biological properties with an exceptional endosomal escape and siRNA delivery efficiency (Gabrielson et al., 2012, Lu et al., 2011, Yin et al., 2013a, Zhang et al., 2011). Nanoparticles have been coated with polyethylene glycol (PEG) (i.e., PEGylated) to enhance their biocompatibility and improve their pharmacokinetics. PEGylated NP with diameters around 100 nm can evade recognition and rapid clearance by macrophages and other elements of the reticuloendothelial system (RES) (Uckun and Yiv, 2012, Davis et al., 2008). We recently reported that the incorporation of PEG further improved the biocompatibility and safety profile of the helical PVBLG-8 block without dramatically compromising the polymer's ability to destabilize membranes (Yin et al., 2013b, Yen et al., 2013). Furthermore, polymer physicochemical properties, especially the 3-dimensional structure, have a significant impact on their membrane penetration and siRNA delivery efficiencies of cationic polypeptides. We recently discovered that a star-shaped PEG-PVBLG-8 copolymer adopting a spherical architecture with high density of PPVLG-8 had the highest membrane-permeabilizing activity and reduced cytotoxicity which contributed to its potent “transfection” efficiency (Yin et al., 2013b). Therefore, we plan to use the star copolymer in preparation of the next generation of PVBLG-8 based nanoparticles. We hypothesize that siRNA nanocomplexes prepared using reconfigured PVBLG-8 building blocks, especially PEG-PVBLG-8 star copolymers with a spherical architecture and high density of PVBLG-8 (Yin et al., 2013b), will exhibit unprecedented in vivo RNAi potency owing to improved serum stability, pharmacokinetic properties, biodistribution and cellular uptake. The development of polypeptide-based multi-functional nanocomplexes with therapeutic siRNA targeting a driver lesion such as the CD22ΔE12-specific siRNA will be a significant step forward to overcome chemotherapy resistance in BPL and other CD22ΔE12^+^ B-lineage lymphoid malignancies.

The expression of CD22 on leukemic B-cell precursors has motivated the development and clinical testing of CD22-directed MoAb, recombinant fusion toxins and antibody-drug conjugates as therapeutic agents against BPL in children (D'Cruz & Uckun, 2013). However, these therapeutic modalities all target the surface epitopes of CD22 and do not discriminate between normal B-cells expressing intact CD22 and BPL cells expressing CD22ΔE12. Due to the presence of CD22 on normal human B-cells and B-cell precursors, lymphotoxicity with reduced B-cell numbers and possible hypogammaglobulinemia with an increased risk of infections would be anticipated side effects of CD22 directed MoAb and MoAb based therapeutics in clinical settings. In contrast, RNAi therapeutics targeting CD22ΔE12 would only kill BPL cells while leaving normal B-cell precursors and B-cells with an intact CD22 encoded by a wildtype CD22 mRNA unharmed.

Our first proof-of-principle showed that the helical polypeptide PVBLG-8 is uniquely suited for preparation of siRNA NP by using the TNFα gene as a target for RNAi (Yin et al., 2013a). Importantly, these helical polypeptide hybrid NP carrying TNFα-specific siRNA displayed membrane-disruptive and endosomolytic properties, did not cause hemolysis at ≤ 50 μg/mL polypeptide concentrations, and exhibited a promising safety profile in mice with no signs of organ specific toxicity in mice at 50 μg/kg (3.5 nmol/kg) or 500 μg/kg (35 nmol/kg) dose levels (Yin et al., 2013a). Based on the recent identification of TNFα as a potentially very important molecular target for RNAi therapy in leukemia (Kagoya et al., 2014), further development of the TNFα-siRNA harboring nanoparticles holds significant translational promise because of their anti-inflammatory, anti-angiogenic, and anti-leukemic potential.

## Authorship contribution

All authors have made significant and substantive contributions to the study. All authors reviewed and revised the paper. F.M.U. was the NIH-funded Principal Investigator who directed and supervised this study, including the preparation of the nanoparticles and wrote the final manuscript. S.Q. performed the bioinformatics and statistical analyses. H.M. performed multiple experiments with siRNA, and PCR and RT-PCR. J.C. and L.Y. provided the polypeptides.

## Conflict of interest disclosure

There are none. The authors declare no competing financial interests.
