# Supplementary material for: A rationally designed nanoparticle for RNA interference therapy in B-lineage lymphoid malignancies
Source: eBioMedicine. 2014 Oct 28;1(2-3):141–55. doi: 10.1016/j.ebiom.2014.10.013 (PMC4292938; doi:10.1016/j.ebiom.2014.10.013)
Supplement: Supplementary file 1 — Supplementary material. [file mmc1.doc]

**SUPPLEMENTAL TABLE AND FIGURES**

**Table S1**. **Signature Phosphoproteome of CD22ΔE12 Transgenic Mouse BPL Cells.**

|  |  | T-test vs. Other | | | |
| --- | --- | --- | --- | --- | --- |
| Protein |  | SD Units |  | (P-value) | Cluster order |
|  |  |  |  |  |  |
| ATPase (Ab-16) |  | 1.41 |  | 0.000880 | 1 |
| mTOR (Ab-2481) |  | 0.98 |  | 0.014379 | 2 |
| ATF2 (Ab-62/44) |  | 1.47 |  | 0.006568 | 3 |
| PKD1/2/3/PKC mu (Ab-744/748) |  | 1.72 |  | 0.000349 | 4 |
| PKD1/PKCmu (Ab-910) |  | 1.55 |  | 0.019585 | 5 |
| p21Cip1 (Phospho-Thr145) |  | 1.57 |  | 0.007637 | 6 |
| P70S6K (Phospho-Thr229) |  | 1.71 |  | 0.007165 | 7 |
| GRK1 (Phospho-Ser21) |  | 1.33 |  | 0.000539 | 8 |
| NFkB-p105/p50 (Ab-932) |  | 1.01 |  | 0.008175 | 9 |
| S6 Ribosomal Protein (Ab-235) |  | 1.32 |  | 0.005596 | 10 |
| Caspase 9 (Ab-144) |  | 1.70 |  | 0.001097 | 11 |
| Tuberin/TSC2 (Phospho-Thr1462) |  | 1.63 |  | 0.005269 | 12 |
| Progesterone Receptor (Ab-190) |  | 1.74 |  | 0.005645 | 13 |
| KSR (Ab-392) |  | 1.96 |  | 0.001205 | 14 |
| AKT (Ab-326) |  | 1.33 |  | 0.012325 | 15 |
| BIM (Phospho-Ser69/65) |  | 1.31 |  | 0.001088 | 16 |
| GAP43 (Phospho-Ser41) |  | 1.39 |  | 0.008900 | 17 |
| GATA1 (Ab-142) |  | 1.60 |  | 0.000561 | 18 |
| JunB (Phospho-Ser259) |  | 1.29 |  | 0.000722 | 19 |
| JunB (Phospho-Ser79) |  | 1.30 |  | 0.018381 | 20 |
| CaMK4 (Ab-196/200) |  | 1.26 |  | 0.004638 | 21 |
| CDC25B (Ab-323) |  | 1.11 |  | 0.017579 | 22 |
| CREB (Ab-133) |  | 1.09 |  | 0.003955 | 23 |
| HSL(Phospho-Ser552/563) |  | 1.73 |  | 0.000005 | 24 |
| FAK (Ab-397) |  | 1.70 |  | 0.014203 | 25 |
| LAT (Ab-171) |  | 1.28 |  | 0.002220 | 26 |
| P38 MAPK (Phospho-Thr180) |  | 1.33 |  | 0.012326 | 27 |
| c-Jun (Ab-170) |  | 1.34 |  | 0.011647 | 28 |
| 14-3-3 zeta (Ab-58) |  | 1.60 |  | 0.017952 | 29 |
| PKC zeta (Ab-410) |  | 0.96 |  | 0.014711 | 30 |
| PLCG2 (Ab-753) |  | 0.99 |  | 0.008524 | 31 |
| ATF-1 (Ab-63) |  | 1.09 |  | 0.005788 | 32 |
| IRS-1 (Ab-636) |  | 1.23 |  | 0.014517 | 33 |
| NFkB-p65 (Ab-435) |  | 1.11 |  | 0.007572 | 34 |
|  |  |  |  |  |  |

Student’s T-tests (Unequal variance correction, Microsoft Excel) compared mean-centered, log_10_ transformed protein expression levels in BPL cells from CD22ΔE12 Tg mice vs. splenocytes from WT mice and BPL cells from BCR-ABL Tg or MYC Tg mice. P-values and effect sizes in SD units are shown for differentially expressed proteins.


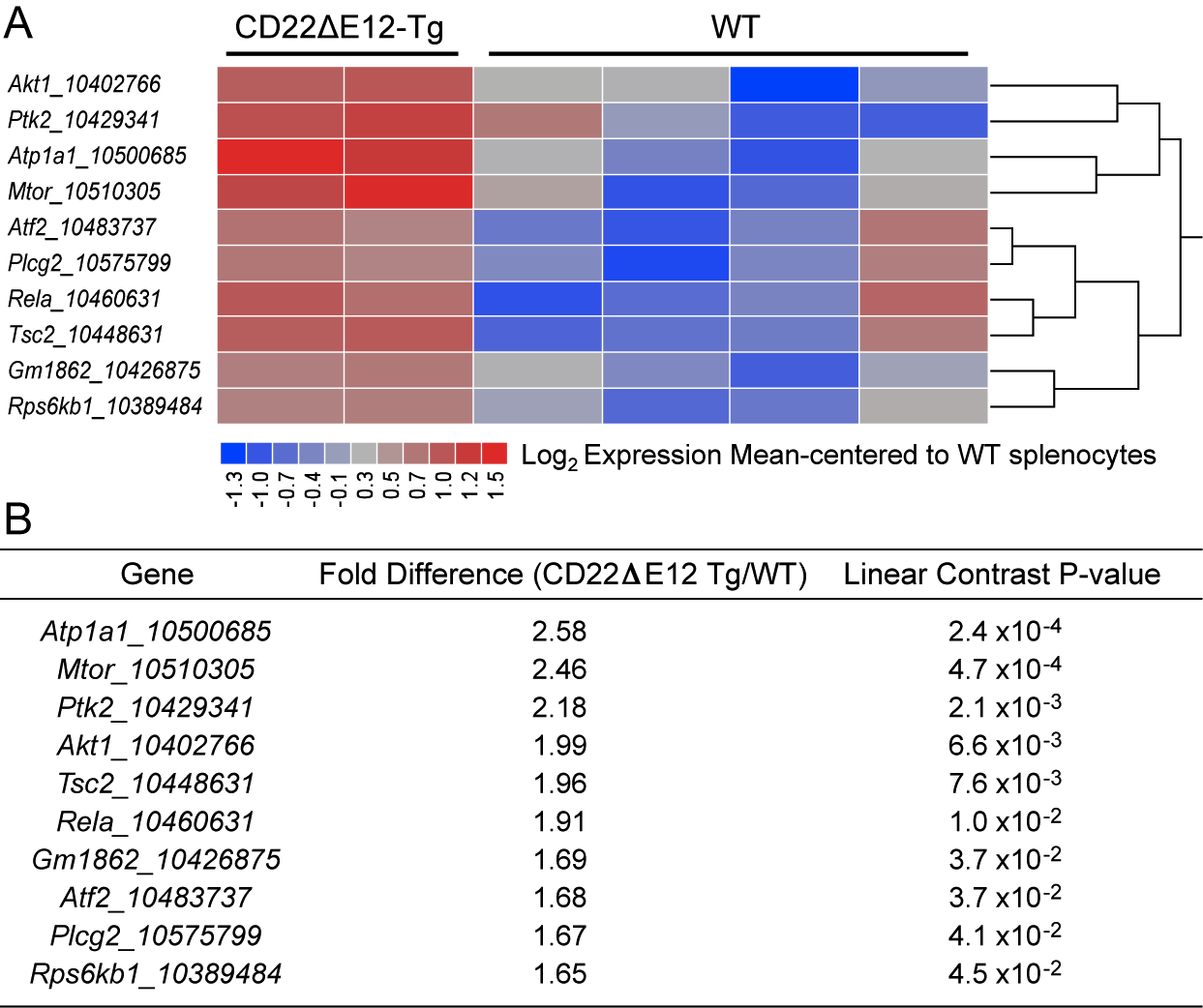


**Figure S1. A CD22ΔE12-Driven Gene Expression Cassette in Mouse BPL Cells.** The gene expression values for splenocytes from WT healthy C57BL/6 mice (N=4), leukemia cells from CD22ΔE12 Tg mice (N=2). Mouse probesets representing the significantly affected proteins identified by the antibody array were compared for differential expression in CD22ΔE12^+^ BPL cells derived from CD22ΔE12-Tg mice (N=2) versus splenocytes of 4 wildtype mice utilizing a Mixed Model to identify significantly affected mRNA levels coding for these proteins (least square means and standard error estimates calculated from the interaction term parameters). Expression levels were estimated utilizing the RMA normalization procedure of signal values obtained using the Affymetrix Mouse Gene 1.0 ST Array platform. Expression values for each probeset were mean centered to WT samples and differences in expression were visualized using a one-way hierarchical cluster figure to organize similar expression profiles of genes across the samples. [A] Heat map depicts up and down regulated transcripts ranging from red to blue respectively and clustered according to average distance metric. [B] Linear contrasts were performed to selectively compare the expression levels of 32 genes (represented by 43 probesets) that encode differentially upregulated phosphoproteins in CD22ΔE12-Tg BPL cells. 10 genes were differentially and significantly upregulated at the mRNA level in CD22ΔE12-Tg BPL cells. Five of these genes (Atp1a1, Mtor, Ptk2, Akt1 and Tsc2) exhibited greater than 1.9 fold increase in CD22ΔE12 Tg mice with P<0.01.


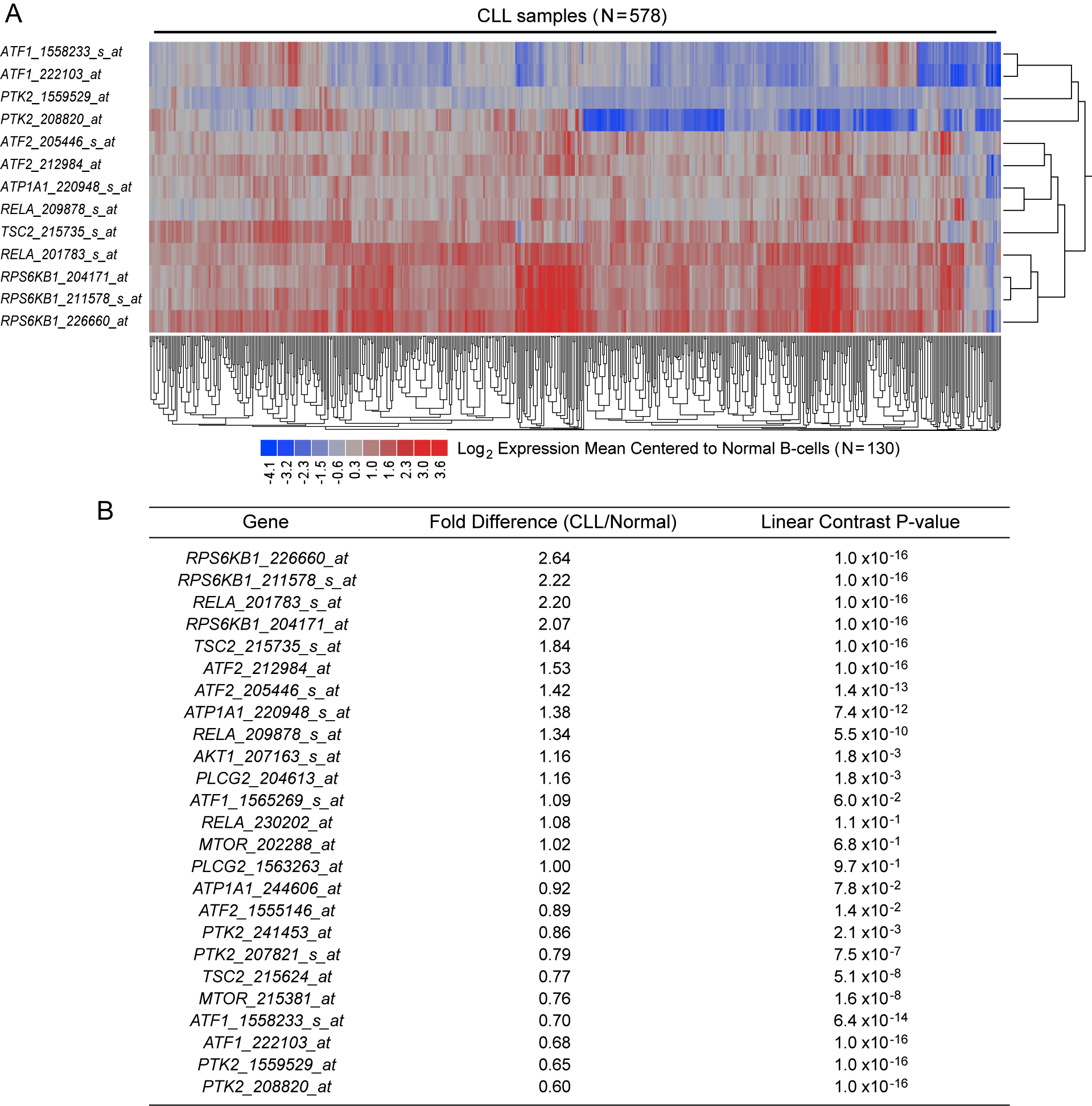


**Figure S2. Upregulated Expression of the** **CD22ΔE12-Driven Gene Expression Cassette in Human Leukemia Cells from Adult Patients with CLL.** The 10-gene mouse CD22ΔE12-Tg gene expression cassette was represented by 25 probesets on the human U133 Plus 2.0 Array. The RMA-normalized gene expression values for leukemia cells from 578 patients with CLL were log_2_ transformed and mean-centered to the average value for the normal B-cells (N=130). **[A]** The gene expression values were clustered according to average distance metric. Heat map depicts up and down regulated transcripts ranging from red to blue respectively for expression values mean centered to normal B-cells in non-leukemic samples. **[B]** To determine the differential expression of each leading edge gene of the CD22ΔE12 transcriptome in CLL cells, linear contrasts were performed for the RMA normalized values (P<0.05 deemed significant).  Mixed Model ANOVA demonstrated significant increases in the multivariate means for the 25 probesets for Normal vs. CLL (Diagnostic group effect F_1,706_ = 15.7, P = 0.00008). Depicted are the mean fold difference and linear contrast P-values relative to normal samples for the comparisons ordered according to effect size. Four transcripts representing 2 genes exhibited greater than 2-fold increases in expression relative to normal (RPS6KB1_226660_at, RPS6KB1_211578_s_at, RELA_201783_s_at, RPS6KB1_204171_at) and 9 transcripts representing 5 genes were highly significantly up regulated (P-value < 1 x 10^-9^, > 1.3 fold increase: ATF2, ATP1A1, RELA, RPS6KB1 and TSC2) as depicted in the cluster figure.


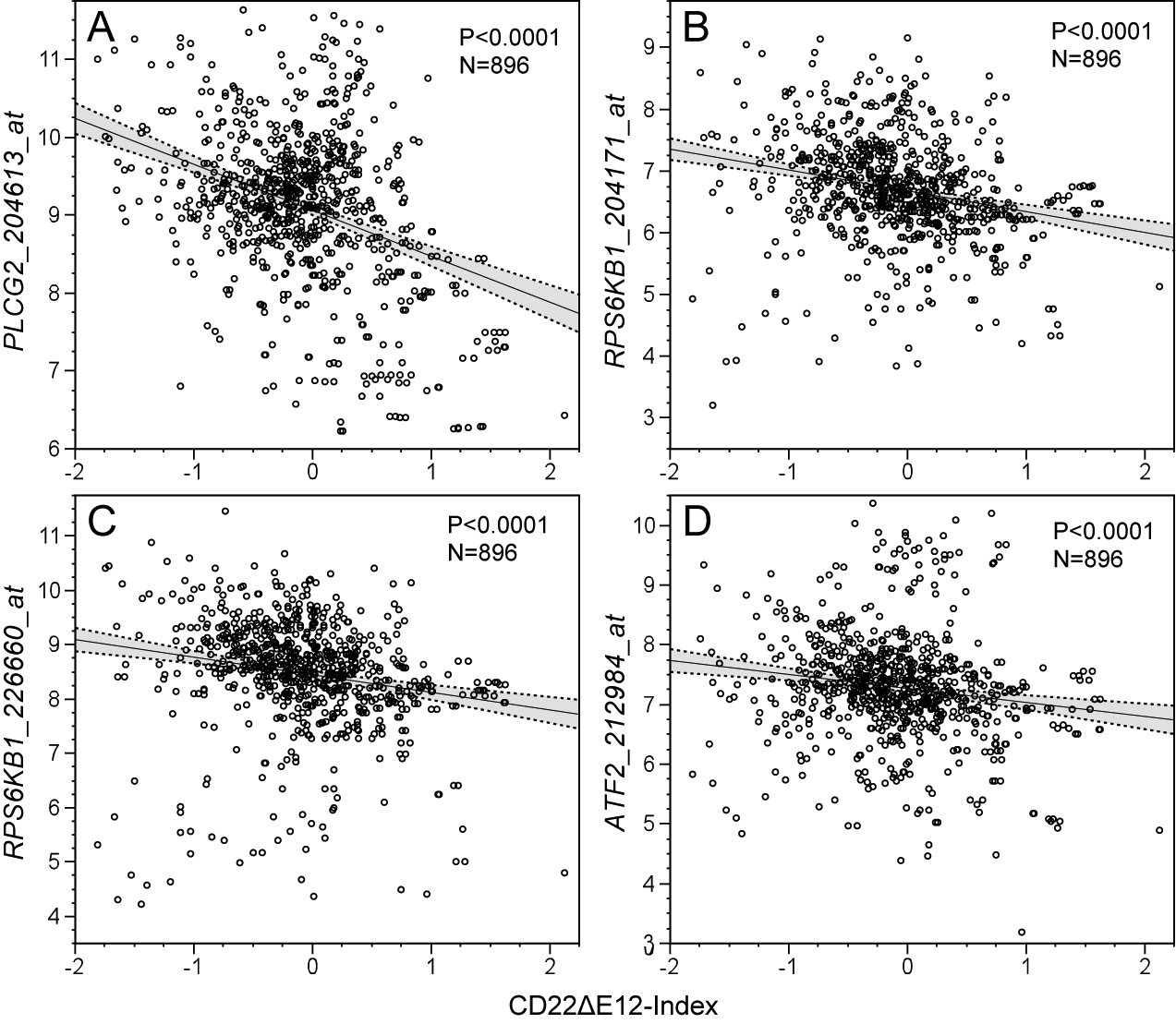


**Figure S3. Correlation between CD22ΔE12-Index and CD22ΔE12-Signature Gene Expression levels in Primary Leukemia Cells.** The CD22ΔE12-driven signature transcriptome differentially expressed in CD22ΔE12 mouse BPL cells included 10 significantly upregulated genes.  This gene expression signature was represented by 25 probesets on the Affymetrix Human Genome U133 Plus 2.0 Array for the human orthologs.  We compared the expression of these genes in normal hematopoietic cells (N=74, GSE13159) versus primary leukemia cells from relapsed BPL patients (N=76, GSE28460, GSE18497), normal lymphoid cells (N=130; GSE10846, GSE12195, GSE12453, GSE13159) versus primary leukemia cells from CLL patients (N=578; GSE13159, GSE39671) and from mantle cell leukemia/lymphoma patients (MCL; N=38, GSE36000) utilizing the RMA normalized database containing these subsets of adult and pediatric leukemias.  Depicted are the bivariate plots of 4 transcripts representing 3 genes that were significantly up regulated across all 3 relapse BPL, CLL and MCL groups of leukemia patients (PLCG2_204613_at, RPS6KB1_204171_at, RPS6KB1_226660_at, ATF2_212984_at) versus CD22ΔE12-Index calculated for each of the samples (N=894 pooled from Normal samples (N=204), CLL samples (N=578), MCL samples (N=38) and relapsed BPL samples (N=76)). Significant negative correlations between CD22ΔE12-index and expression of each of the 4 probesets (PLCG2_204613_at, r = -0.35, F_1,894_ = 124, P<0.0001; RPS6KB1_204171_at, r = -0.22, F_1,894_ = 47, P<0.0001; RPS6KB1_226660_at, r = -0.18, F_1,894_ = 28, P<0.0001; ATF2_212984_at, r = -0.14, F_1,894_ = 17, P<0.0001) were observed suggesting that a loss of expression of CD22 Exon12 results in the upregulation of the mouse gene signature genes.


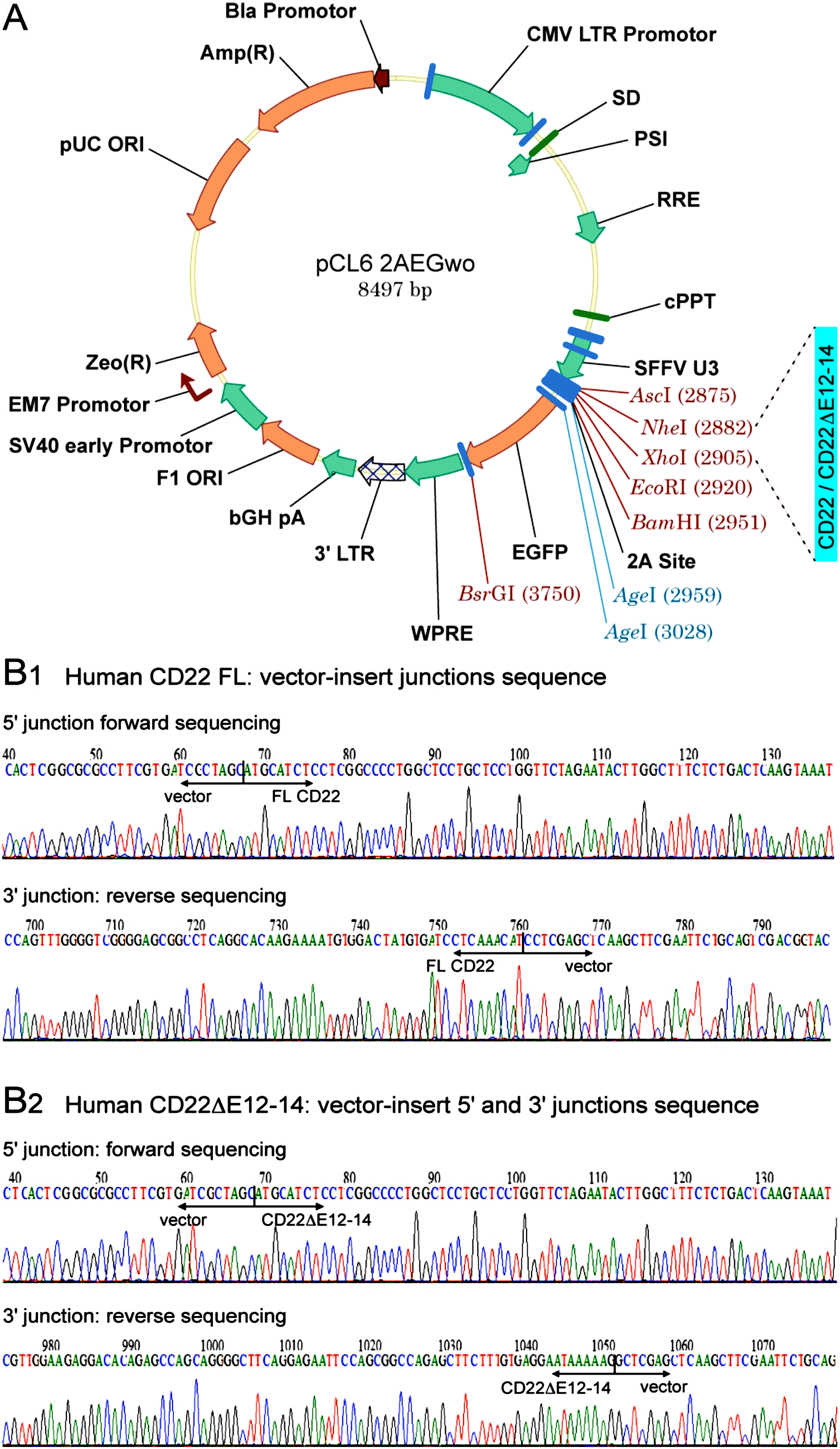


**Figure S4. Lentiviral Construct with CD22ΔE12-14 Insert. [A]** Depicted is a plasmid map of the lentiviral vector (pCL6-2AEGwo) that was used to clone human CD22ΔE12-14 to generate a FISH probe for the CD22ΔE12-transgene. This vector contains a 2A site (APVKQTLNFDLLKLAGDVESNPGP) and an in-frame eGFP-coding fragment downstream of a multiple cloning site. **[B]** Shown are the sequencing chromatograms depicting the sequence of the CD22 FL (B1) and CD22ΔE12-14 (B.2) inserts and vector junctions at both the 5’ and 3’ ends of the inserts. The Sanger sequencing method was used by GENEWIZ, Inc. (South Plainfield, NJ) using Applied Biosystems BigDye version 3.1, and the reactions were run on an Applied Biosystem's 3730xl DNA Analyzer with the forward primer 5-CAGCCTGCTTCTCGCTTCTGTT-3’ and the reverse primer 5’-CTCCTGCCAACTTGAGAAGGTC-3’. The obtained DNA sequences were analyzed by BioEdit v7.2.0 (<http://www.mbio.ncsu.edu/bioedit>).


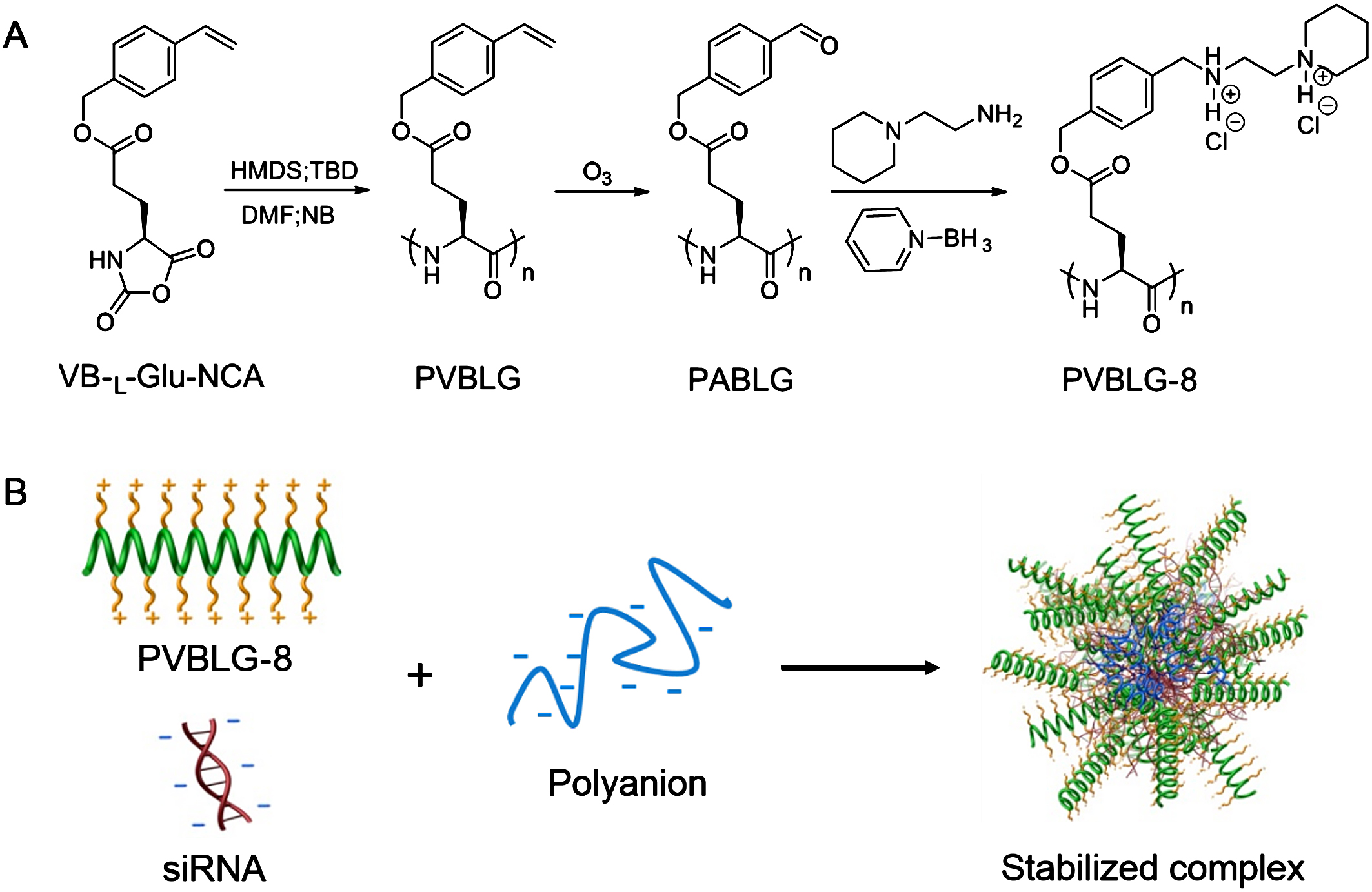


**Figure S5. [A] Reaction scheme of PVBLG-8. (B) Schematic representation of PVBLG-8/polyanion/siRNA complex**
